# Maturation of circulating Ly6C^hi^CCR2^+^ monocytes by mannan-MOG induces antigen-specific tolerance and reverses autoimmune encephalomyelitis

**DOI:** 10.3389/fimmu.2022.972003

**Published:** 2022-09-09

**Authors:** Anastasia Dagkonaki, Athina Papalambrou, Maria Avloniti, Areti Gkika, Maria Evangelidou, Maria-Eleni Androutsou, Theodore Tselios, Lesley Probert

**Affiliations:** ^1^ Laboratory of Molecular Genetics, Department of Immunology, Hellenic Pasteur Institute, Athens, Greece; ^2^ Department of Chemistry, University of Patras, Patras, Greece; ^3^ Research and Development Department, Vianex PLC, Nea Erythrea, Greece

**Keywords:** demyelination, immunotherapy, monocyte maturation, PD-L1, Cre/loxP mouse, multiple sclerosis, MDSC, EAE

## Abstract

Autoimmune diseases affecting the CNS not only overcome immune privilege mechanisms that protect neural tissues but also peripheral immune tolerance mechanisms towards self. Together with antigen-specific T cells, myeloid cells are main effector cells in CNS autoimmune diseases such as multiple sclerosis, but the relative contributions of blood-derived monocytes and the tissue resident macrophages to pathology and repair is incompletely understood. Through the study of oxidized mannan-conjugated myelin oligodendrocyte glycoprotein 35-55 (OM-MOG), we show that peripheral maturation of Ly6C^hi^CCR2^+^ monocytes to Ly6C^hi^MHCII^+^PD-L1^+^ cells is sufficient to reverse spinal cord inflammation and demyelination in MOG-induced autoimmune encephalomyelitis. Soluble intradermal OM-MOG drains directly to the skin draining lymph node to be sequestered by subcapsular sinus macrophages, activates Ly6C^hi^CCR2^+^ monocytes to produce MHC class II and PD-L1, prevents immune cell trafficking to spinal cord, and reverses established lesions. We previously showed that protection by OM-peptides is antigen specific. Here, using a neutralizing anti-PD-L1 antibody *in vivo* and dendritic cell-specific *Pdl1* knockout mice, we further demonstrate that PD-L1 in non-dendritic cells is essential for the therapeutic effects of OM-MOG. These results show that maturation of circulating Ly6C^hi^CCR2^+^ monocytes by OM-myelin peptides represents a novel mechanism of immune tolerance that reverses autoimmune encephalomyelitis.

## Introduction

Immune-mediated demyelinating diseases such as multiple sclerosis (MS) are characterized by infiltration of the CNS by T and B lymphocytes and myeloid cells, which co-operate to induce inflammation in the white and grey matter and progressive neuronal damage (1). Autoimmune demyelination is best modelled by experimental autoimmune encephalitis (EAE), a disease in which myelin antigen-specific T cells are both critical and sufficient to initiate pathology, supporting the concept that MS is an autoimmune disease (2, 3). However, the direct targeting of T cells has shown little therapeutic success in MS (4). Myeloid cells predominate in demyelinated areas of CNS in both EAE and MS, and infiltrating monocytes are the main effector cells in disease initiation and demyelination (5–9). In EAE, most CNS-infiltrating mononuclear cells are Ly6C^hi^CCR2^+^ inflammatory monocytes that expand in the blood and spleen from bone marrow myeloid precursors following immunization, are highly mobile and cross the blood-brain barrier into the CNS in a CCR2-dependent manner disease (8, 10–13). Once in the CNS, they upregulate MHC class II molecules (MHCII), the proinflammatory signature enzyme inducible nitric oxide synthase (iNOS), and inflammatory mediators such as TNF and IL-1β, and then differentiate into monocyte-derived effector macrophages and dendritic cells (DC) during EAE (10, 14–16). It is these CNS-infiltrating monocytes, not resident microglia, that are essential for the effector phase of EAE (8). The differentiation of Ly6C^hi^ monocytes into monocyte-derived effector macrophages and DC within the CNS is instructed by interferon-γ (IFNγ) and granulocyte-macrophage colony-stimulating factor (GM-CSF) produced by infiltrating activated Th1 and Th17 cells, respectively (17–19). However, the mechanisms of immune tolerance that normally retain these cells in the periphery and protect against CNS autoimmunity are not well understood.

Bone marrow derived myeloid precursor cells massively expand in the periphery under conditions of chronic inflammation, cancer, infection and autoimmunity. In the context of cancer they have been named myeloid-derived suppressor cells (MDSC) based on their immunosuppressive activities (20). Using phenotypic criteria, mouse and human MDSC can be separated into two major subgroups, namely monocytic MDSC (M-MDSC) and granulocyte/polymorphonuclear MDSC (G-MDSC) based upon the cell lineage from which they originate. In EAE and MS the roles of MDSC appear to be complex, involving both proinflammatory and immunosuppressive effects. In MS, increased frequencies of circulating M-MDSC and G-MDSC were observed in relapse-remitting MS patients before relapse compared to those with stable disease (21). In EAE, M-MDSC (CD11b^+^Ly6C^high^Ly6G^-^, equivalent to Ly6C^hi^ cells) and G-MDSC (CD11b^+^Ly6G^+^Ly6C^low^, here called Ly6G^+^ cells) accumulate in bone marrow, spleen, blood (12, 22), lung (23), and spinal cord (24, 25) after immunization and, as mentioned above, pathogenic inflammatory Ly6C^hi^ monocytes are critical for disease (10). Spleen and lung Gr1^+^ myeloid cells (containing both Ly6C^hi^ and Ly6G^+^ populations) promote Th17 cell differentiation and exacerbate EAE (26, 27). On the other hand, Ly6C^hi^ and Ly6G^+^ splenocytes isolated from EAE immunized mice displayed T cell suppressive MDSC effects *in vitro* (12), and *in vivo* when adoptively transferred into recipient mice during EAE (22), respectively. Indeed, infiltrating Ly6C^hi^CCR2^+^ monocytes show high plasticity in the CNS during EAE and become increasingly activated, undergoing monocyte-to-phagocyte transition and switching from Ag presentation to T cell suppression *via* production of nitric oxide and the induction of T cell apoptosis (15, 19, 28), or polarizing to beneficial arginase-producing cells involved in debris removal and tissue repair (16). Ly6G^+^ cells also differentiate into MDSC within the CNS during EAE, and these cells inhibit B cell proliferation *in vitro* (25). Recently, a novel population of CXCL10-expressing phagocytes with different origin to Ly6C^hi^ cells, was isolated from EAE spinal cord, suggesting that additional myeloid cell populations might contribute to disease pathogenesis (29). Understanding the contributions of peripheral Ly6C^hi^CCR2^+^ monocytes to CNS autoimmune disease, and the mechanisms that control their switching between pathogenic and beneficial effects, is important for the design of novel immunotherapies for MS.

We recently showed that myelin antigens conjugated to an oxidized form of fungal mannan polysaccharide (OM) induce antigen-specific immune tolerance and are potent inhibitors of autoimmune demyelination when administered intradermally (i.d.) in mice as prevention or treatment (30). Tolerance involves a peripheral type 2 myeloid cell response and some features of anergy in antigen-specific T cells (31). Fungal mannan is a pattern recognition receptor (PRR) ligand that binds the mannose receptor expressed mainly on cells of the myeloid lineage, induces the maturation of DC *ex vivo* in a TLR4-dependent manner, and acts as an innate immune adjuvant (32–34). Until now, the observation by us and others that OM-conjugated (30) and mannosylated (35) myelin antigens induce potent antigen-specific immune tolerance in EAE has therefore appeared paradoxical and been difficult to reconcile with the immune adjuvant effects of mannan. To help resolve this question, here we investigated whether immature Ly6C^hi^ and Ly6G^+^ myeloid cells are involved in immune tolerance induced by OM-conjugated myelin oligodendrocyte glycoprotein peptide 35-55 (OM-MOG) in EAE. Using the chemotherapeutic drug gemcitabine (GEM), which selectively depletes MDSC in tumor-bearing animals (36), we confirm that highly proliferating Ly6C^hi^MHCII^-^ myeloid cells are critical for the onset of EAE, and show that their induction is not required for OM-MOG immune tolerance. Instead, soluble OM-MOG drains directly to the skin draining lymph node (DLN) and activates Ly6C^hi^CCR2^+^ monocytes to produce MHCII and PD-L1, thereby blocking their migration to the CNS to induce disease. We further show that PD-L1 production by cells other than dendritic cells (DC), including Ly6C^hi^ and Ly6C^lo^ cells, is sufficient to mediate the therapeutic effects of OM-MOG in EAE. Notably, a single therapeutic intradermal i.d. injection of OM-MOG is sufficient to reduce inflammatory spinal cord lesions in mice with active EAE, suggesting that peripheral activation of Ly6C^hi^CCR2^+^ myeloid progenitor cells might be a promising way forward to achieve selective immunotherapy in autoimmune diseases affecting the human CNS.

## Materials and methods

### Mice

C57BL/6 mice (B6; Harlan) and CD45.1 congenic B6 mice (kindly provided by Burkhard Becher and Melanie Greter, University of Zurich), *Pdl1ff*, and *Cd11c*-Cre (both lines kindly provided by Arlene Sharpe, Harvard University) (37), were maintained under specific pathogen-free conditions in the Department of Animal Models for Biomedical Research of the Hellenic Pasteur Institute. All experimental procedures were reviewed and approved by the Committee for Evaluation of Experimental Procedures of the Institute (presided over by Dr P. Andriopoulos, pandriopoulos@patt.gov.gr, for the Hellenic Republic, General Secretariat for Agricultural Economy, Veterinary and Licenses), license numbers 2580/29-05-2018 and 662033/05-08-2021, conformed to ARRIVE guidelines and were in compliance with national PD 56/2013 and European Directive 2010/63/EU for the use of non-human animals for scientific purposes.

### Synthesis of peptide conjugates and administration protocols

Mouse MOG35-55 (MOG) and MOG with a (KG)5 peptide linker were synthesized in solid phase by 9-fluorenylmethylocarboxyl/t-butoxy (Fmoc/tBu) methodology using Fmoc protected amino acids, 2-chlorotrityl chloride resin, and standard N,N’diisopropyl carbodiimide/1-hydrobenzotriazole coupling agents, and (KG)5-MOG was conjugated to oxidized mannan (OM) as previously described (38, 39). OM-MOG was administered to mice using prophylactic and therapeutic protocols. In a long-term prophylactic protocol, groups of 6- to 8-week-old female B6 mice were injected i.d. on the hind flanks with 100μl of OM-MOG (30 μg MOG equivalent/injection and 700μg OM equivalent/injection), or vehicle (carbonate-bicarbonate buffer pH 9.0). Three consecutive injections were performed with 15-day intervals, and EAE was induced 15 days after the last injection. In a short-term prophylactic protocol, groups of 8-week-old female B6 mice received 3 consecutive i.d. OM-MOG injections starting just before the onset of clinical symptoms on day post immunization (dpi) 7, and thereafter dpi 11 and 14. In the therapeutic (treatment) protocol, mice received i.d. OM-MOG injections starting when individual mice reached score 2 and thereafter every 2 days, or alternatively every 3 days in the experiments involving administration of anti-PD-L1 neutralizing antibody or mice with CD11c^+^ DC-specific deletion of PD-L1 (dcPD-L1KO mice).

OM-FITC-β-Ala-[KG]5MOG35-55 (Ser42) peptide (OM-F-MOG) was synthesized as follows: (KG)5-MOG was synthesized and conjugated with FITC after the removal of Fmoc group by the N-terminal amino acid (β-Ala) (see **Supplementary Material**). The crude deprotected peptide analogue was purified by semi-preparative reverse phase high performance liquid chromatography (RP-HPLC) and identified by electron spray ionization mass spectrometry (ESI-MS). The purity of the final product was verified by RP-HPLC (see **Supplementary Material**). The conjugation of F-MOG with mannan was achieved as for (KG)5-MOG above (38, 39).

### EAE induction and treatments

MOG-EAE was induced in groups of 8-12-week-old or 12-14-week-old (after long-term prophylactic treatment protocol) female B6 mice by immunization with s.c. tail-base injection of 38μg MOG in 100μl saline emulsified in equal volume of CFA (Sigma-Aldrich). CFA was supplemented with 400μg/injection of H37Ra *Mycobacterium tuberlculosis* (BD Biosciences). Immunization with CFA without MOG was performed as adjuvant control. All mice received i.p. injections of 200 ng of *Bordetella pertussis* toxin (PTx) (Sigma-Aldrich) at the time of immunization and 48 h later. Mice were monitored daily for the clinical symptoms of EAE according to the following clinical scores: 0, normal, 1, limp tail, 2, hind limb weakness, 3, hind limb paralysis, 4, forelimb paralysis, 5, moribund or dead (0.5 gradations represent intermediate scores). Animals from score 4 upwards were euthanized. All mice were allowed free access to food and water throughout the experiments. GEM was administered to mice using prophylactic and therapeutic protocols. In the prophylactic protocol, mice were injected i.p. with 60 mg/kg GEM in saline, before the onset of clinical symptoms of EAE at dpi 6 and 10. In one experiment shown (**Figure 2C**), an additional injection was made after clinical onset at dpi 17. In the therapeutic protocol, mice were injected i.p. with 60 mg/kg GEM in saline, every 2 days, starting when individual mice reached score 2 for a total of 6 injections. Depletion of myeloid cell populations by GEM was monitored in samples of peripheral blood (PB) collected from the tail vein, or in splenocytes collected at sacrifice, by flow cytometry. Neutralizing anti-CD274/PD-L1 monoclonal antibody (clone 10F.9G2, Biolegend catalog 124338) was administered (i.p 200μg/mouse/injection) in groups of mice with ongoing EAE receiving therapeutic injections of OM-MOG or vehicle, 24 h prior to each OM-MOG/vehicle injection. Mice received a total of three consecutive injections of anti-CD274 antibody with 3-day intervals, as described above for OM-MOG in this experiment.

### T cell proliferation assay

Splenocytes were isolated from EAE mice at sacrifice. Spleens were dissociated through a 70 μm cell strainer to prepare single-cell suspensions in RMPI 1640 (Invitrogen Life Technologies) containing 10% heat-inactivated FCS and erythrocyte lysis was performed with Gey’s erythrocyte lysis buffer, as previously described (31). Washed splenocytes (10^7^ cells/ml in PBS) were labeled with carboxylfluorescein succimidyl ester (CFSE) (CFDA-SE; Thermofisher) at a final concentration of 5 μM for 15 min at 37°C. Cells were washed and incubated with PBS/2% FCS for 30 min at 37°C to stop the reaction. Cells were washed and resuspended in RPMI 1640/FCS containing 50 μm 2-mercaptoethanol (Sigma-Aldrich) at 10^6^ cells/ml and stimulated in duplicate in round-bottom 96-well plates with 15 μg/ml MOG peptide for 96 h. Control cells were stimulated with medium alone as negative control or with plate bound anti-CD3 (0.5μg/ml) (clone 145-2C11, BD Biosciences) as positive control. Results are expressed as division index, which is the average number of cell divisions that a cell in the original population has undergone and was calculated as the total number of divisions divided by the total number of cells at the start of culture.

### Adoptive transfer of MOG-stimulated splenocytes and DLN cells into EAE recipient mice

Groups of 6-8-week-old CD45.1 congenic mice were injected with OM-MOG or vehicle using the long-term prophylactic protocol described above. Both CD45.1 congenic donor mice and naïve B6 (CD45.2) recipient mice were immunized for EAE. Splenocytes and DLN cells were recovered from CD45.1 donors on dpi 9 just prior to the onset of clinical symptoms, pooled, stimulated overnight *ex vivo* with MOG peptide (10μg/ml) and stained with CFSE as described above. CD45.1 cells were transferred (i.p. 10 x10^6^/mouse) into CD45.2 recipient EAE mice on dpi 10. Splenocytes and CNS-infiltrating mononuclear cells were recovered from the CD45.2 recipients on dpi 14 for flow cytometry analysis.

### Isolation of CNS-infiltrating mononuclear cells

CNS-infiltrating mononuclear cells were isolated separately from preparations of spinal cord meninges and spinal cord without meninges dissected from EAE mice at sacrifice on dpi 14 (at mean clinical score 2). Briefly, whole vertebral columns were opened by dorsal laminectomy and the leptomeninges were removed from the spinal cord using a stereoscope and fine forceps. Leptomeninges were placed in a petri dish containing 2mM EDTA and 5mM Hepes (Gibco, 15630080) in HBSS (w/o CaCl_2_ or MgCl_2_, Gibco, 14175053) for 1 h at 4°C, massaged using the rubber plunger from a 1ml syringe and the buffer containing the cells collected. Mononuclear cells were isolated from the remaining spinal cord by Percoll gradient centrifugation, for flow cytometric analysis, as previously described (40).

### Flow cytometry

Splenocytes were isolated as described above. Peripheral blood mononuclear cells (PBMC) were isolated from 200μl tail vein blood samples from EAE mice by 3-4 consecutive washes with ACK erythrocyte lysis buffer. For cell surface marker staining, cells were fixed in 2% paraformaldehyde in PBS (PFA) for 20 min at 4°C and immunostained using fluorochrome-conjugated antibodies to B220-APC (RA3-6B2, Biolegend), CD4-APC/Cy7 (clone RM4-5; BD Biosciences), CD11b- APC or FITC (clone M1/70, BD Biosciences), CD11c-FITC (clone HL3, BD Biosciences), CD11c-PerCP (clone N418, Biolegend), CD45.1-PE (clone A20, BD Biosciences), CD45.2-APC (clone 104, BD Biosciences), Ly6C-APC (clone AL-21, BD Biosciences), Ly6G-PE (clone 1A8, BD Biosciences), I-A/I-E-FITC (clone 2G9, BD Biosciences), PD-L1-PE (clone MIH5, BD Biosciences), and Annexin V-FITC (BD Biosciences), for 30 min at 4°C. For CCR2 cell surface staining live cells were incubated with fluorochrome-conjugated antibody to CCR2-PE (clone SA203G11, Biolegend), for 15 min at 37°C and for additional 15 min at RT. For intracellular staining of cytokines, cells where treated with phorbol 12-myristate 13-acetate (PMA) (10 ng/ml, Sigma-Aldrich) and ionomycin (1μg/ml, Sigma-Aldrich) in the presence of brefeldin-A (5μg/ml, Sigma-Aldrich) for 3 h at 37°C/5% CO_2,_ fixed with 2% PFA as above, permeabilized with 0.5% wt/vol saponin, and stained with fluorochrome-conjugated antibodies to CD4-FITC (clone GK1.5, Biolegend), IFN-γ-PE (clone XMG1.2; BD Biosciences), IL-17A-APC (clone TC11-18H10.1, Biolegend) and arginase 1-PE/Cy7 (clone A1exF5, Thermofisher). Data was acquired using a FACSCalibur or FACSAria cytometer and analyzed with FlowJo software (Tree Star, Inc).

### Histopathology and immunofluorescence staining

Mice were transcardially perfused with ice-cold 4% PFA at sacrifice by carbon dioxide inhalation. DLN and other lymph nodes were dissected and post-fixed in the same fixative overnight. The vertebral column was dissected and post-fixed in the same fixative for 48 h at 4°C. For histopathology, spinal cord was removed, embedded in paraffin and 5 μm paraffin sections processed for standard histopathological analysis. Inflammation was visualized by haematoxylin and eosin (H&E) and demyelination by Luxol fast blue (LFB)/periodic acid-Schiff staining. Sections were observed using an Olympus BX-50 microscope and images captured with an Olympus DP71 microscope digital camera using cell^A imaging software (Soft Imaging System GmbH). Inflammation was determined by semi-quantitative scoring as follows: 1: foci of subarachnoid cell infiltration or meningeal inflammation, 2: diffuse subarachnoid infiltration or perivascular infiltration, 3: foci of parenchymal infiltration, 4: diffuse widespread parenchymal infiltration. Demyelination was evaluated by semi-quantitative scoring as follows; 0.5: single perivascular sleeves of demyelination, 1: ubiquitous perivascular or sub-pial demyelination, 2: confluent demyelinated plaques, 3: profound focal demyelination, involving about 1/2 of the spinal cord white matter at least in one spinal cord segment, 4: extensive demyelination, for instance complete demyelination of spinal cord white matter in one or more segment of cord. Images were scored blindly by two independent observers.

For immunofluorescence staining of DLN from mice injected with OM-F-MOG, or as antibody-binding control non-fluorescence OM-MOG, the tissues were equilibrated with 15% and 30% sucrose overnight each, mounted in optimal temperature compound (OCT) and snap frozen in melting isopentane. Longitudinal 20μm cryostat sections were mounted on microscope slides for immunofluorescence staining. Sections were incubated with PBS containing 10% FCS and 0.5% Triton X-100 (Sigma-Aldrich) for 1 h at RT to block non-specific staining, followed by primary antibody rat anti-CD169 mAb (1:150, 3D6.112, Biolegend) overnight at 4°C, followed by secondary antibody CF647 goat anti-rat IgG (1:2000, Biotium: 20283) for 1 h at RT. Nuclei were counterstained DAPI (Invitrogen; catalog D1306). Immunofluorescence was observed and captured using a Leica TCS-SP8 MP confocal microscope. All images were acquired as stacks of 12-14 slices imaged at 2.0-μm depth intervals using 10x, 20x and 63x oil immersion objectives.

For immunofluorescence staining of whole mount spinal cord sections, the vertebral column was post-fixed and decalcified with 10% EDTA for 10 days at RT, equilibrated with 15% and 30% sucrose overnight each, mounted in OCT and snap frozen in melting isopentane. Transverse 40 μm cryostat sections were mounted on microscope slides for immunofluorescence staining. Sections were incubated with PBS containing 10% FCS and 0.5% Triton X-100 (Sigma-Aldrich) for 1 h at RT to block non-specific staining, followed by primary antibodies rat anti-CD45 mAb (1:200, 30-F11, Biolegend), rabbit anti-pan-laminin (455, kindly provided by Lydia Sorokin, University of Muenster), and rabbit anti-myelin basic protein (MBP) (1:200, Abcam, ab40390) overnight or 48 h (MBP) at 4°C, followed by secondary antibodies AlexaFluor 568 anti-rabbit IgG (1:1000 Invitrogen; A11011) and AlexaFluor- 488 anti-rat IgG (1:1000, Invitrogen; A11006) for 1 h or overnight (MBP) at RT. Nuclei were counterstained DAPI (Invitrogen; catalog D1306). Immunofluorescence was observed and captured using a Leica TCS-SP8 MP confocal microscope. All images were acquired as stacks of 20-22 slices imaged at 2.0-μm depth intervals using 10x and 40x oil immersion objectives.

### Statistical analysis

All statistical analysis was performed with Graphpad Prism 8 and Microsoft Excel software. All data are given as mean ± SEM and were tested for normality using the Shapiro-Wilk test. Data showing normal distribution were analyzed using Student’s t test for pairwise comparisons between groups, and Student’s t test followed by Bonferroni *post hoc* test or one-way ANOVA followed by Tukey’s *post hoc* test for multiple comparisons between groups. Non parametric data from EAE scoring of mice were analyzed at each time point by the Mann-Whitney test. Results were considered statistically significant when p ≤ 0.05.

## Results

### Ly6C^hi^ myeloid cells are selectively depleted from the periphery during the acute phase of EAE

**Figure 1:**
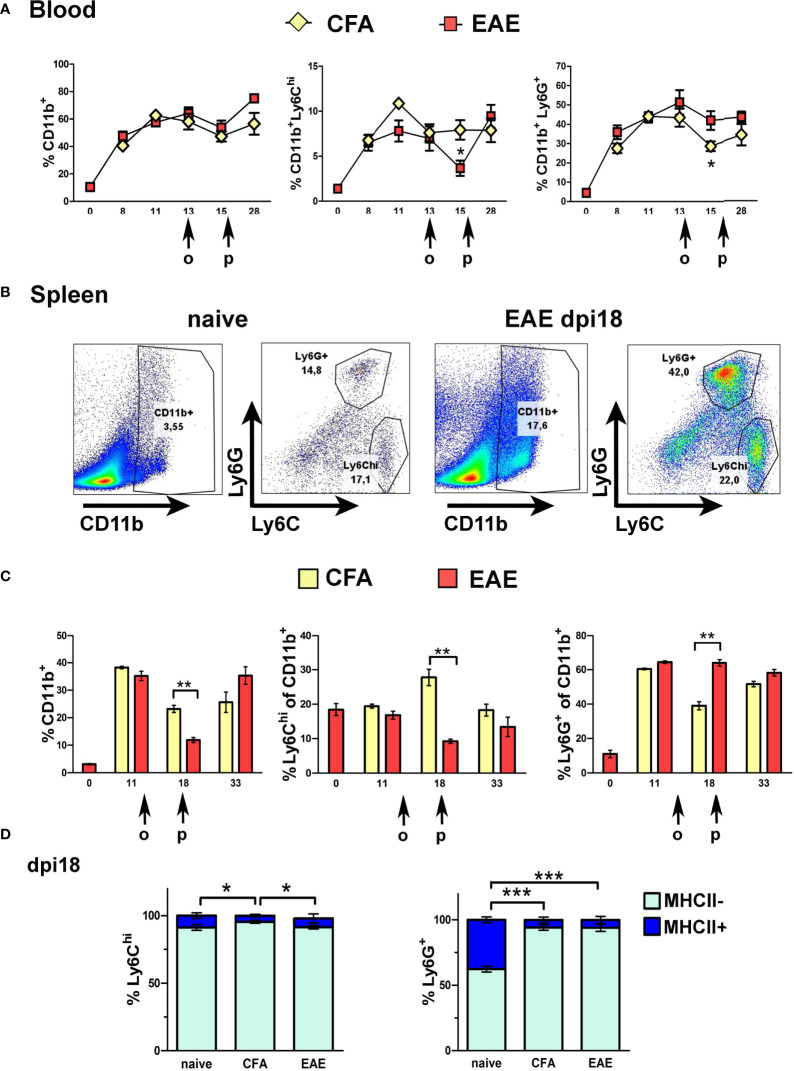
Ly6C^hi^ and Ly6G^+^ myeloid cells expand in the blood and spleen in response to immunization. **(A)** Time-course of CD11b^+^, Ly6C^hi^ and Ly6G^+^ myeloid cell expansion in blood of CFA and EAE immunized mice (n=2 at day 0 and n=4-5/group/time-point). Arrows indicate the days of onset (o) and peak (p) of clinical symptoms in the EAE mice. **(B)** Gating strategy of CD11b^+^ sub-populations in spleens of naïve and EAE mice at the peak of clinical symptoms (dpi 18). **(C)** Time-course of CD11b^+^, Ly6C^hi^ and Ly6G^+^ myeloid cell expansion in spleens of CFA and EAE immunized mice (n=3-4/group/time-point). Arrows indicate the days of onset (o) and peak (p) of clinical symptoms in the EAE mice. **(D)** Proportions of MHCII^-^ and MHCII^+^ splenocytes within the Ly6C^hi^ and Ly6G^+^ subpopulations in CFA and EAE immunized mice at the peak of clinical symptoms (dpi 18) (n=3/group). Data and statistical analysis are derived from one experiment in each case (one for **A**, **C**, one for **B**, **D**). Statistical significance is shown after pairwise comparisons between groups using Student’s t test **(A, C)** or multiple comparisons using one-way ANOVA followed by Tukey’s *post hoc* test **(D)** (*p ≤ 0.05, **p ≤ 0.01, ***p ≤ 0.001).

The expansion of CD11b^+^ cells and the main immature myeloid cell subpopulations that express Gr1, Ly6C^hi^ and Ly6G^+^ cells, was measured in peripheral blood (PB) and spleen of mice before (naïve) and after immunization with MOG for the induction of EAE, or without MOG as adjuvant control (CFA). CD11b^+^ cell expansion was equal in PB of EAE and CFA mice at all time points corresponding to pre-onset (dpi 8 and 11), onset (dpi 13), peak (dpi 15-18) and chronic phase (dpi 28) of clinical symptoms in the EAE group (**Figure 1A**). Ly6C^hi^ and Ly6G^+^ subpopulations also showed equal expansion in PB of EAE and CFA mice except for dpi 15 (peak), where proportions of Ly6C^hi^ monocytes were significantly decreased in EAE PB corresponding to their migration into the CNS during disease (**Figure 1A**). Myeloid cell expansion was reflected in the spleen, where CD11b^+^ cells were equally increased in EAE and CFA mice pre-onset (dpi 11) and chronic phase (dpi 33) of symptoms in the EAE group compared to naïve mice (**Figure 1B** showing flow cytometry gating strategy; **Figure 1C**). Again, Ly6C^hi^ cells were significantly reduced, as were total CD11b^+^ cells, in the spleen of EAE mice compared to CFA mice at dpi 18 (peak), corresponding to their migration into the CNS (**Figure 1C**). In contrast to Ly6C^hi^ cells, proportions of Ly6G^+^ cells were not depleted in PB or spleen during EAE. These findings are consistent with previous reports that CD11b^+^Gr1^+^ cells expand in bone marrow, PB and spleen of mice immunized with MOG for the induction of EAE (12, 22). The finding of sharply reduced peripheral Ly6C^hi^ cells in EAE compared to CFA mice at the peak of disease is also consistent with previous reports that these cells migrate to the spinal cord during the symptomatic phases of EAE (8).

Consistent with the immature status of peripheral Ly6C^hi^ and Ly6G^+^ cells, most of these cells in spleen of naïve and immunized mice were MHCII^-^ (**Figure 1D**). Like CD11b^+^ and Ly6C^hi^ cells, Ly6C^hi^MHCII^-^ cells, and not Ly6G^+^MHCII^-^ cells, showed a significant reduction in the periphery at EAE peak compared to CFA, consistent with selective migration of Ly6C^hi^ monocytes into the CNS during EAE (**Figure 1D**). Arginase 1 (Arg1), an enzyme involved in metabolism of L-arginine and a marker for tumor-associated G-MDSC (20), and alternative “M2” macrophages (41), was produced at high levels by splenic Ly6G^+^ cells but not by Ly6C^hi^ cells, further consistent with a role of Ly6C^hi^ monocytes in pathology (**Supplementary Figure 1A**).

### Gemcitabine transiently depletes EAE-induced peripheral Ly6C^hi^ and Ly6G^+^ cells, delays disease onset and does not break OM-MOG-induced immune tolerance

**Figure 2:**
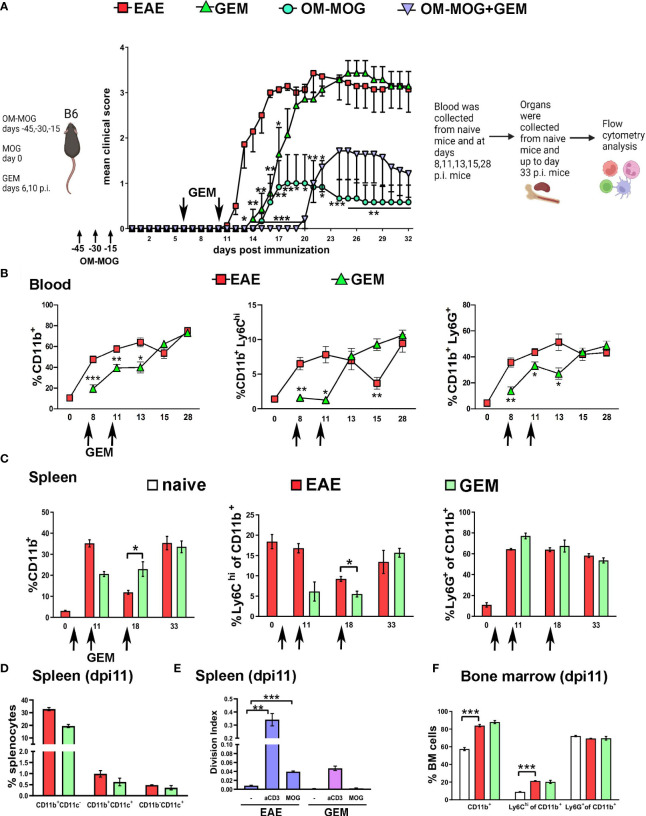
Gemcitabine transiently delays EAE onset and extends protection by OM-MOG. **(A)** Schematic representation of the experimental approach and the time points of injection of OM-MOG and gemcitabine (GEM) in the EAE mice. Mean clinical scores of EAE mice after long-term prophylactic administration of OM-MOG (days -45, -30, -15 relative to immunization for EAE, upward arrows) and pre-onset administration of GEM (dpi 6 and 10, downward arrows) (n=6-7/group). **(B)** Time-course of CD11b^+^, Ly6C^hi^ and Ly6G^+^ myeloid cell expansion in blood of EAE mice untreated or treated with GEM (dpi 6 and 10; arrows) (n=2 at day 0 and n=4-5 mice/group/time-point). **(C)** Time-course of CD11b^+^, Ly6C^hi^ and Ly6G^+^ myeloid cell expansion in spleens of EAE mice untreated or treated with GEM (dpi 6, 10 and 17; arrows) (n=2-3/group/time-point). **(D)** Proportions of different populations of myeloid cells, including CD11b^+^CD11c^-^, CD11b^+^CD11c^+^ (myeloid DC) and CD11b^-^CD11c^+^ DC cells, in spleens of EAE mice untreated or treated with GEM shown in C, at dpi 11 (n=2-3/group). **(E)** T cell proliferation responses to MOG or anti-mouse CD3e in spleens of EAE mice untreated or treated with GEM shown in C, at dpi 11 (n=2-3/group). Data are expressed as division index (total number of divisions/cells at start of culture). **(F)** Proportions of CD11b^+^, Ly6C^hi^ and Ly6G^+^ myeloid cells in bone marrow of EAE mice untreated or treated with GEM shown in C, at dpi 11, and naïve mice (n=2-3/group). Data and statistical analysis are derived from one experiment in each case (one for **A**, one for **B–F**). Statistical significance is shown after comparisons to the vehicle (EAE) group using Mann-Whitney test **(A)**, or pairwise comparisons between groups using Student’s t test **(B–D, F)** or Student’s t test followed by Bonferroni *post hoc* test **(E)** (*p ≤ 0.05, **p ≤ 0.01, ***p ≤ 0.001).

To investigate the functional involvement of Ly6C^hi^ and Ly6G^+^ cells in MOG-EAE we first used the chemotherapeutic drug GEM. GEM is a deoxycytidine analog that inhibits ribonucleotide reductase and selectively depletes CD11b^+^Gr1^+^ myeloid cells in tumor-bearing animals where these cells are immunosuppressive, and thereby enhances anti-tumor immune responses (36). To minimize potential effects on T cell activation and proliferation (26), GEM (i.p. 60 mg/Kg) was first administered in groups of OM-MOG- and vehicle-treated EAE mice on dpi 6 and 10 close to disease onset (**Figure 2A**). Pre-onset GEM administration significantly delayed the onset of clinical symptoms but did not prevent the subsequent development of full-blown disease (**Figure 2A**).

To confirm that GEM inhibits the expansion of Ly6C^hi^ and Ly6G^+^ cells, we performed flow cytometry in PB samples at different time points after EAE immunization (**Figure 2B**) and in splenocytes recovered at sacrifice (**Figure 2C**). Pre-onset GEM administration transiently depleted blood CD11b^+^ (dpi 8, 11, 13), Ly6C^hi^ (dpi 8, 11) and Ly6G^+^ (dpi 8, 11, 13) cells, corresponding to the delay in EAE onset in GEM-treated mice (**Figure 2A**). Interestingly, blood Ly6C^hi^ monocytes sharply increased in GEM-treated mice at dpi 13 to levels equal to those in non-treated EAE mice (**Figure 2B**), and this corresponded to the disease onset in GEM mice (**Figure 2A**). CD11b^+^ cell populations were equal in GEM and non-treated EAE mice in the chronic phase of disease (dpi 28; **Figure 2B**). Results in PB were reflected in spleen, with GEM mice showing a tendency to reduced CD11b^+^ and Ly6C^hi^ cells at dpi 11 (**Figures 2C, D**), corresponding to the delay in EAE onset in GEM-treated mice (**Figure 2A**). We also monitored the effects of GEM on other immune cell populations in the spleen of EAE mice. In contrast to Ly6C^hi^ and Ly6G^+^ cells, DC (CD11b^+^CD11c^+^ and CD11b^-^CD11c^+^ cells), were not altered by GEM at dpi 11, a time point corresponding to markedly reduced Ly6C^hi^ monocytes and delayed disease onset in GEM mice (**Figure 2D**). Also, proportions of CD4^+^ T cells, Th1 and Th17 cells were not altered by GEM at dpi 11 (**Supplementary Figure 1B**). However, consistent with a previous report (26), MOG-specific and polyclonal T cell proliferation responses that are typical in splenocytes from EAE mice were reduced by GEM at this time point (**Figure 2E**). GEM treatment did not alter the expansion of CD11b^+^ myeloid cell populations induced by EAE immunization in the bone marrow at dpi 11 (**Figure 2F**).

We next tested whether immature CD11b^+^ myeloid cells positively contribute to immune tolerance induced by OM-MOG. Consistent with our previous reports (30, 31), prophylactic administration of OM-MOG protected mice against EAE (**Figure 2A**). OM-MOG protection is characterized by reduced antigen-specific T cell proliferation responses without alterations in effector T cell maturation or survival (30), and associated with a peripheral type 2 myeloid cell response (31). To investigate whether OM-MOG induces immune tolerance by induction of Ly6C^hi^ or Ly6G^+^ cells with MDSC properties, we administered GEM to mice after administration of OM-MOG using the long-term prophylactic protocol and immunization for EAE. GEM given prior to disease onset did not prevent and even extended protection by prophylactic OM-MOG, further supporting a pathogenic function of Ly6C^hi^ cells in EAE (**Figure 2A**).

In summary, GEM transiently reduced proliferation of Ly6C^hi^, Ly6G^+^ myeloid cells and T cells in immunized mice, and administration in EAE mice prior to disease onset delayed clinical symptoms. Ly6C^hi^ monocytes were depleted from PB at clinical peak in both EAE (dpi15, blood) and GEM-treated EAE (dpi18, spleen) mice corresponding to their known migration to the CNS to induce disease (8). Prophylactic OM-MOG protection against EAE was maintained and extended by GEM administration, suggesting that OM-MOG immune tolerance is not mediated by Ly6C^hi^ cells with MDSC properties.

### OM-MOG prevents EAE by retaining Ly6C^hi^ cells in the periphery

**Figure 3:**
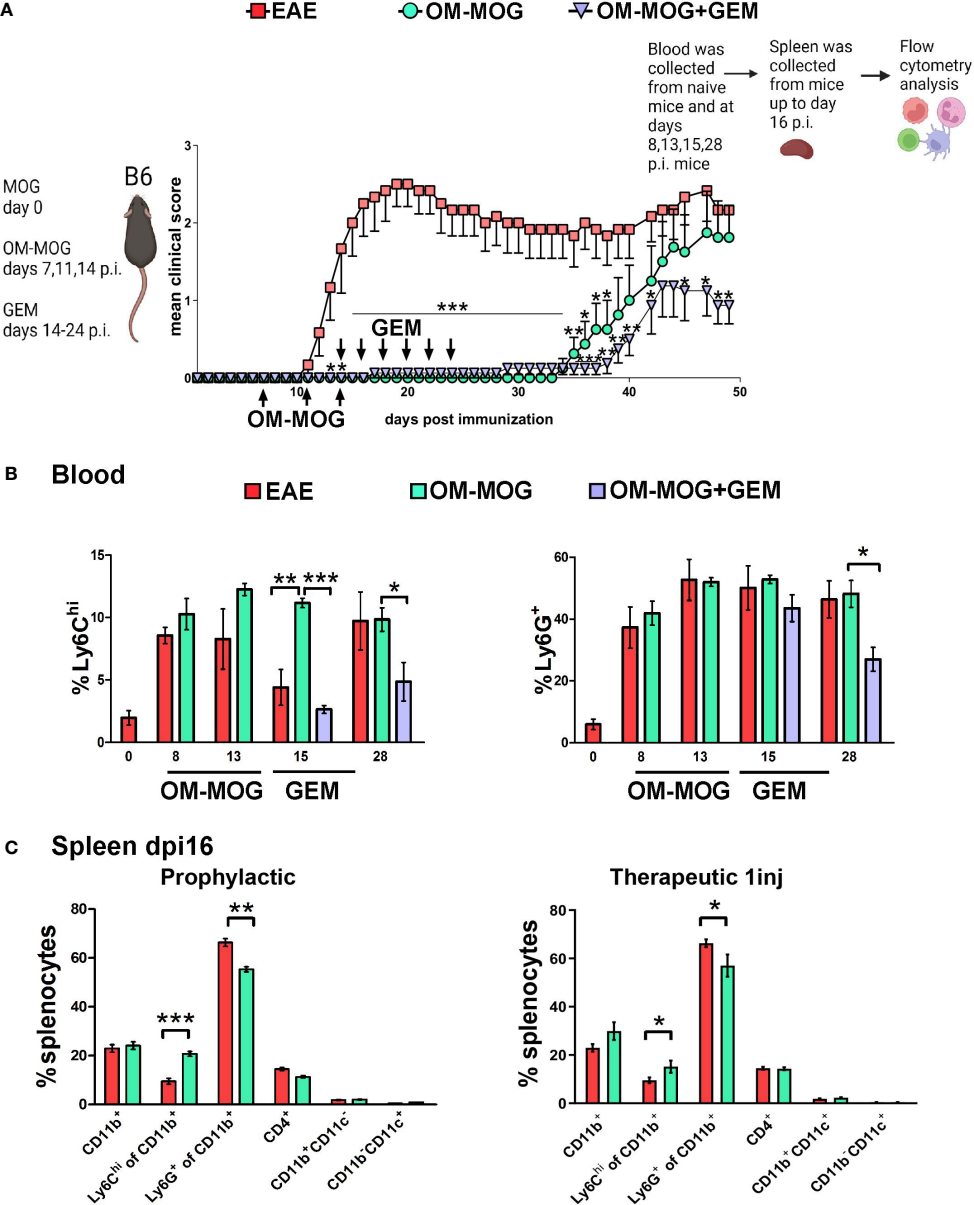
OM-MOG retains Ly6C^hi^ monocytes in the periphery during the induction of EAE. **(A)** Schematic representation of the experimental approach and the time points of injection of OM-MOG and gemcitabine (GEM) in the EAE mice. Mean clinical scores of EAE mice after short-term prophylactic administration of OM-MOG (dpi 7, 11 and 14, upward arrows) and post-onset administration of GEM (dpi 14 and every 2 days, downward arrows) (n=6-8/group). **(B)** Time-course of Ly6C^hi^ and Ly6G^+^ cell expansion in blood of EAE mice untreated or treated with GEM, as shown in A (n= 3-4/group/time-point). **(C)** Proportions of CD11b^+^, Ly6C^hi^, Ly6G^+^, CD11b^+^CD11c^+^ (myeloid DC) and CD11b^-^CD11c^+^ (DC) myeloid cells and CD4^+^ T cells in spleen of EAE mice after short-term prophylactic (as shown in A., left panel) or therapeutic (24 h post-injection, right panel) administration of vehicle or OM-MOG, at peak of disease in the vehicle mice (dpi 16) (n=8 for EAE and n=4 for OM-MOG). Data and statistical analysis are derived from one experiment in each case (one for **A, B**, one for **C**). Statistical significance is shown after comparison to the vehicle (EAE) group using Mann-Whitney test **(A)**, one-way ANOVA followed by Tukey’s *post hoc* test **(B)**, or Student’s t test **(C)** (*p ≤ 0.05, **p ≤ 0.01, ***p ≤ 0.001).

To exclude effects of GEM on the onset of EAE and examine more clearly its effects on OM-MOG tolerance, we next administered it therapeutically after EAE onset. Mice were injected with OM-MOG using the short-term prophylactic protocol between dpi 7-14 and subsequently with GEM between dpi 14-24. As expected, OM-MOG mice were strongly protected against the clinical symptoms of EAE for up to 20 days after the last injection (**Figure 3A**). Consistent with the effects of prophylactic GEM, therapeutic GEM extended protection by OM-MOG (**Figure 3A**).

Myeloid cell depletion by GEM after EAE immunization was monitored longitudinally in PB samples at different time points (**Figure 3B**) and in splenocytes recovered from mice sacrificed at dpi 16 (**Figure 3C**). Therapeutic OM-MOG did not alter the expansion of Ly6C^hi^ and Ly6G^+^ cells in PB following immunization. Importantly, OM-MOG completely prevented the depletion (migration) of Ly6C^hi^ monocytes from the blood at dpi 15, which characterizes disease initiation in EAE mice (**Figure 3B**), and this was reflected by the absence of disease in OM-MOG EAE mice (**Figure 3A**). GEM efficiently depleted Ly6C^hi^ monocytes in PB of OM-MOG EAE at dpi 15, and of both Ly6C^hi^ and Ly6G^+^ cells on dpi 28, confirming these cells are highly proliferating (**Figure 3B**), and this correlated with extended protection against disease by OM-MOG (**Figure 3A**). OM-MOG also prevented selective depletion of Ly6C^hi^, not Ly6G+, cells from the spleen at dpi 16 (EAE peak) following short-term prophylactic (**Figure 3C**, left panel) or, in a separate experiment, even a single therapeutic injection (**Figure 3C**, right panel). To understand whether OM-MOG administration results in the accumulation of cells in the periphery, we compared spleen cellularity in EAE and OM-MOG-treated EAE mice in the chronic phase (dpi 33). Prophylactic OM-MOG increased total numbers of splenocytes and CD4^+^ T cells, and tended to increase numbers of CD11b+ myeloid cells compared to control EAE mice (**Supplementary Figure 1C**). Together, these results provide the first indication that OM-MOG ameliorates EAE by preventing the migration of pathogenic Ly6C^hi^ cells from the periphery to the CNS.

### OM-MOG prevents trafficking of immune cells to spinal cord meninges and parenchyma

**Figure 4:**
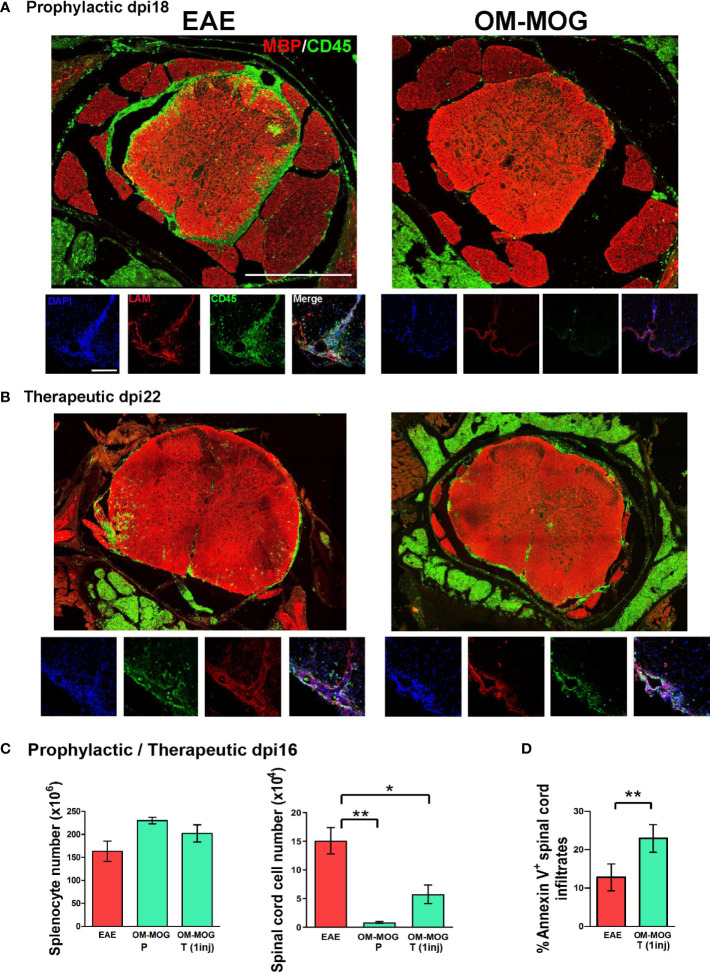
OM-MOG prevents and reverses immune cell infiltration and demyelinating lesions in the spinal cord during EAE. **(A)** Whole mount spinal cord sections with surrounding meninges and vertebrae prepared from EAE mice after short-term prophylactic administration of vehicle or OM-MOG (dpi 7, 11 and 14) at peak of disease in the EAE group (dpi 18), and immunostained with anti-MBP for myelin (red) and anti-CD45 for immune infiltrates (green) (low power photomicrographs), or anti-laminin for blood brain barrier (LAM; red), anti-CD45 (green) and DAPI (blue) (high power photomicrographs). Representative images from 1 of 3 mice/group. Scale bars 500 μM (upper low-power images) and 100 μM (lower high-power images). **(B)** Whole mount spinal cord sections prepared from mice with ongoing EAE (dpi 22) after therapeutic treatment with 4 injections of vehicle or OM-MOG starting at clinical score 2, and immunostained with anti-MBP for myelin (red) and anti-CD45 for immune infiltrates (green) (low power magnification), or anti-laminin for blood brain barrier (LAM; red), anti-CD45 (green) and DAPI (blue) (high power magnification). Representative images from 1 of 3 mice/group. **(C)** Total numbers of splenocytes and CNS-infiltrating mononuclear cells recovered from spinal cords of EAE mice at disease peak (dpi 16) after short-term prophylactic administration, as in A (OM-MOG P), or 24 h after 1 therapeutic injection of vehicle or OM-MOG (OM-MOG T, 1 inj) (n=8 for EAE and n=4 for OM-MOG). **(D)** Proportions of Annexin V^+^ CNS- infiltrating mononuclear cells recovered from spinal cords of EAE mice at disease peak (dpi 14) 24 hours after 1 therapeutic injection of vehicle (EAE) or OM-MOG (OM-MOG T, 1 inj) (n=4-5/group). Data and statistical analysis are derived from one experiment in each case **(A–C)** or one representative of two experiments **(D)**. Statistical significance is shown after comparison to the vehicle (EAE) group using one-way ANOVA followed by Tukey’s *post hoc* test **(C)**, or Student’s t test **(D)** (*p ≤ 0.05, **p ≤ 0.01).

To investigate whether OM-MOG inhibits EAE by blocking immune cell trafficking from the periphery to the CNS, or by preventing immune cell infiltration across the blood brain barrier, we performed immunohistochemical analyses of whole-mount vertebral column sections in which the structure of spinal cord, leptomeninges and surrounding tissues was fully preserved. First, mice were treated with OM-MOG using the short-term prophylactic protocol, immunized for EAE and sacrificed for analysis on dpi 18, corresponding to EAE peak in the vehicle group (see **Figure 2A**). Immunostaining with the pan-leukocyte marker CD45 showed prominent inflammation of the leptomeninges and immune cell infiltration of the spinal cord parenchyma in EAE mice (**Figure 4A**, left photograph). In contrast, few immune cells were found in the vicinity of the spinal cord in OM-MOG mice (**Figure 4A**, right photograph). We interrogated the integrity of meningeal and blood brain barriers by double immunostaining sections with antibodies to CD45 and laminin, a major component of the basement membrane that is involved in blood brain barrier regulation (42). Spinal cord lesions in EAE mice were characterized by severe barrier disruption and immune cell infiltration of parenchymal tissues, while OM-MOG mice showed preserved barrier integrity with scarce associated immune cells (**Figure 4A**, high power photographs).

The finding that prophylactic OM-MOG inhibited the migration of CD45-immunoreactive immune cells to the spinal cord was further supported by the results of adoptive transfer experiments using CD45.1 congenic mice. Here, CD45.1 “donor” splenocytes and LN cells isolated from congenic mice after prophylactic injection with OM-MOG or vehicle and immunization for EAE, were adoptively transferred into wild-type (CD45.2) B6 recipient mice that had been similarly immunized for EAE and compared for recruitment to CNS tissues by flow cytometry (**Supplementary Figure 2A**). CNS-infiltrating mononluclear cells were recovered separately from meninges and spinal cord parenchyma at different time points from onset to peak and analyzed for cell surface CD45.1 and CD45.2 by flow cytometry. As expected, CD45.1^+^ cells from prophylactic OM-MOG donor mice showed significantly reduced recruitment to spinal cord meninges compared to CD45.1^+^ cells from vehicle-treated donor mice (**Supplementary Figure 2B**).

We next treated EAE mice with 4 therapeutic injections of OM-MOG (every 2 days starting on dpi 14) and analyzed spinal cord pathology at sacrifice on dpi 22. Inflammatory spinal cord lesions in therapeutic OM-MOG EAE mice were significantly reduced compared to those in vehicle EAE mice, with infiltrating CD45-immunoreactive immune cells being mainly localized to the meninges, not parenchyma, as defined by laminin immunostaining (**Figure 4B**, high power photographs). Counting of immune cells in mice from equivalent groups of mice at dpi 16 (EAE peak) showed significantly reduced CNS-infiltrating mononuclear cells in the spinal cord, remarkably as early as 24 h after a single therapeutic injection of OM-MOG, accompanied by a tendency for increased total numbers of splenocytes (**Figure 4C**). The reduction of CNS-infiltrating mononuclear cells in the spinal cord of mice treated by a single injection of OM-MOG was accompanied by increase of cell surface Annexin V staining in the remaining cells (**Figure 4D**). These results show that OM-MOG prevents trafficking of encephalitogenic immune cells from the periphery to the spinal cord, accumulation in the subarachnoid space and infiltration into the CNS parenchyma to induce EAE (prophylactic effect) or to perpetuate ongoing EAE (therapeutic effect). The reduction of CNS-infiltrating mononuclear cells in the spinal cord after a single therapeutic injection of OM-MOG is associated with increased apoptosis in the residual immune infiltrates.

### OM-MOG rapidly activates peripheral Ly6C^hi^CCR2^+^ monocytes to produce MHCII and PD-L1

**Figure 5:**
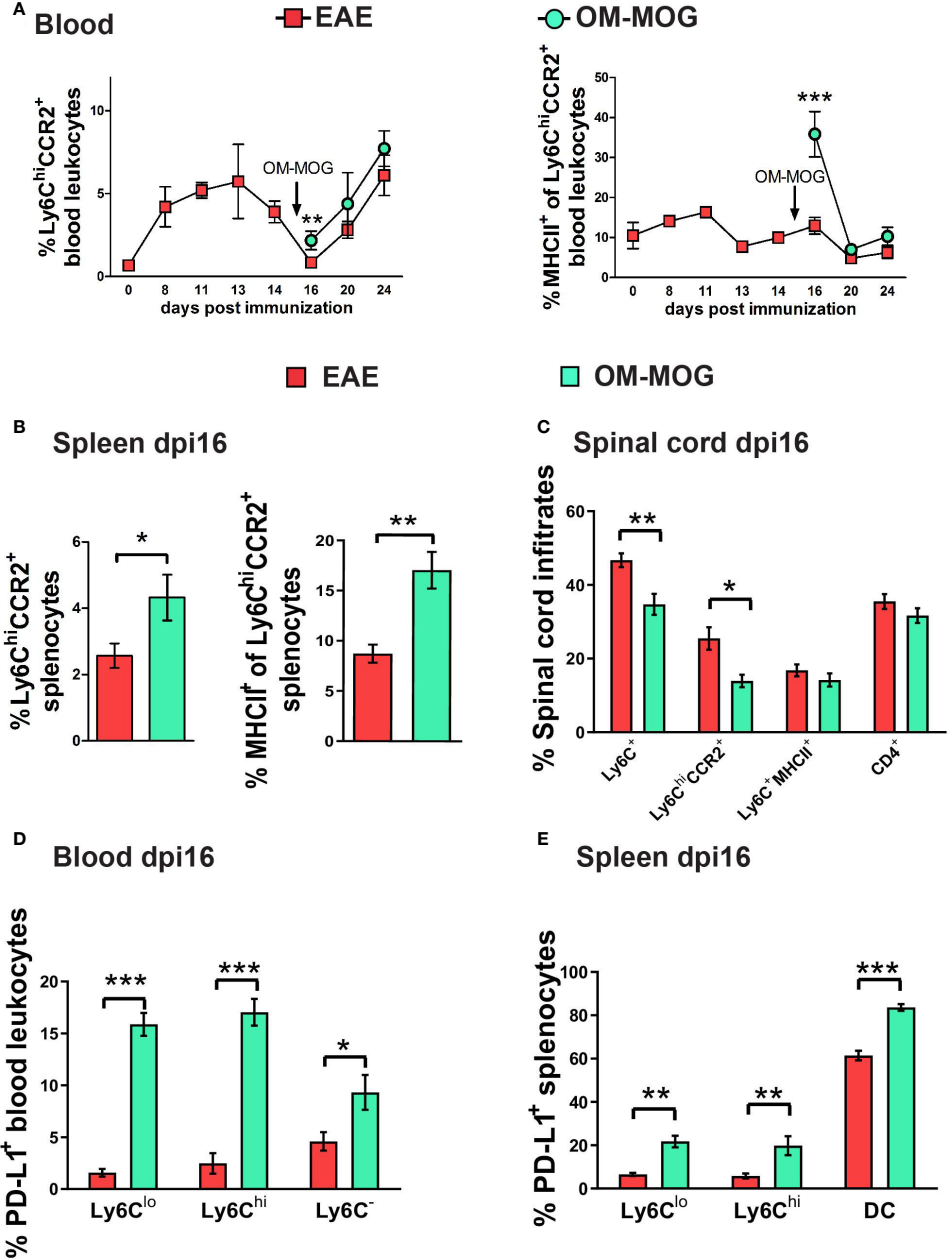
Therapeutic OM-MOG alternatively activates and retains Ly6C^hi^CCR2^+^ monocytes in the periphery of EAE mice, while inhibiting infiltration of the spinal cord. **(A)** Time-course of proportions of Ly6C^hi^CCR2^+^ monocytes (left graph) and Ly6C^hi^CCR2^+^ monocytes expressing MHCII (right graph) in blood of EAE mice treated therapeutically with vehicle or OM-MOG from dpi 15 (arrow) every two days (n=3 at day 0 and n=3-10/group/time-point). **(B)** Proportions of Ly6C^hi^CCR2^+^ monocytes (left graph) and Ly6C^hi^CCR2^+^ monocytes expressing MHCII (right graph) in spleen of EAE mice at disease peak (dpi 16) 24 hours after 1 therapeutic injection of vehicle (EAE) or OM-MOG 15 (arrow in **A**). **(C)** Proportions of Ly6C^hi^CCR2^+^ and Ly6C^+^MHCII^+^ cells and CD4^+^ T cells in CNS-infiltrating mononuclear cells recovered from spinal cord of mice shown in A at disease peak (dpi 16) 24 hours after 1 therapeutic injection of vehicle (EAE) or OM-MOG (n=8 for EAE and n=4 for OM-MOG). Proportions of PD-L1-producing, **(D)** CD11c^-^Ly6C^lo^, CD11c^-^Ly6C^hi^ myeloid cells and Ly6C^-^ cells in blood (n=6 for EAE and n=4 for OM-MOG), and **(E)** CD11c^-^Ly6C^lo^, CD11c^-^Ly6C^hi^ myeloid cells and CD11c^+^Ly6C^-^ DC in spleen (n=8 for EAE and n=4 for OM-MOG) of mice shown in A at disease peak (dpi 16) 24 hours after 1 therapeutic injection of vehicle (EAE) or OM-MOG. Data and statistical analysis are from one experiment. Statistical significance is shown after pairwise comparisons between groups using Student’s t test (*p ≤ 0.05, **p ≤ 0.01, ***p ≤ 0.001).

Production of CCR2 by Ly6C^hi^ cells is essential for their exit from bone marrow (43) and migration to the CNS and development of EAE (11, 13), so we first tested whether OM-MOG regulates CCR2 production by peripheral Ly6C^hi^ cells. Proportions of Ly6C^hi^CCR2^+^ monocytes in PB during EAE development (**Figure 5A**) mirrored those of Ly6C^hi^ monocytes (**Figure 1A**), showing peak expansion at disease onset (dpi 13) and depletion at clinical peak (dpi 16). A single therapeutic injection of OM-MOG increased proportions of Ly6C^hi^CCR2^+^ cells, and sharply increased MHCII production by Ly6C^hi^CCR2^+^ cells, after 24 h in PB and spleen of dpi 16 EAE mice (clinical peak) (**Figures 5A, B**). Similarly, OM-MOG markedly increased PD-L1 production by Ly6C^hi^ cells, Ly6C^lo^ cells which are likely derived from Ly6C^hi^ precursors (44), and Ly6C^-^ cells in PB, and by Ly6C^hi^ and Ly6C^lo^ cells and DC in spleen after 24 h (**Figures 5D, E**). Conversely, CNS-infiltrating mononuclear cells contained reduced proportions of Ly6C^hi^ and Ly6C^hi^CCR2^+^ cells and low levels of Ly6C^hi^MHCII^+^ cells (**Figure 5C**). Short-term prophylactic OM-MOG similarly increased Ly6C^hi^CCR2^+^ cells in the periphery and reduced their infiltration into spinal cord, and increased PD-L1 production by Ly6C^lo^ cells in PB and spleen (**Supplementary Figure 3**). So far, these results show that OM-MOG does not alter CCR2 production by peripheral Ly6C^hi^ cells, and that even a single injection of soluble OM-MOG is sufficient to induce MHCII and PD-L1 production by circulating Ly6C^hi^ monocytes and to prevent their migration to the spinal cord to induce EAE.

### Soluble OM-MOG drains directly to skin DLN to locally activate PD-L1- and MHCII-producing Ly6C^hi^ cells

**Figure 6:**
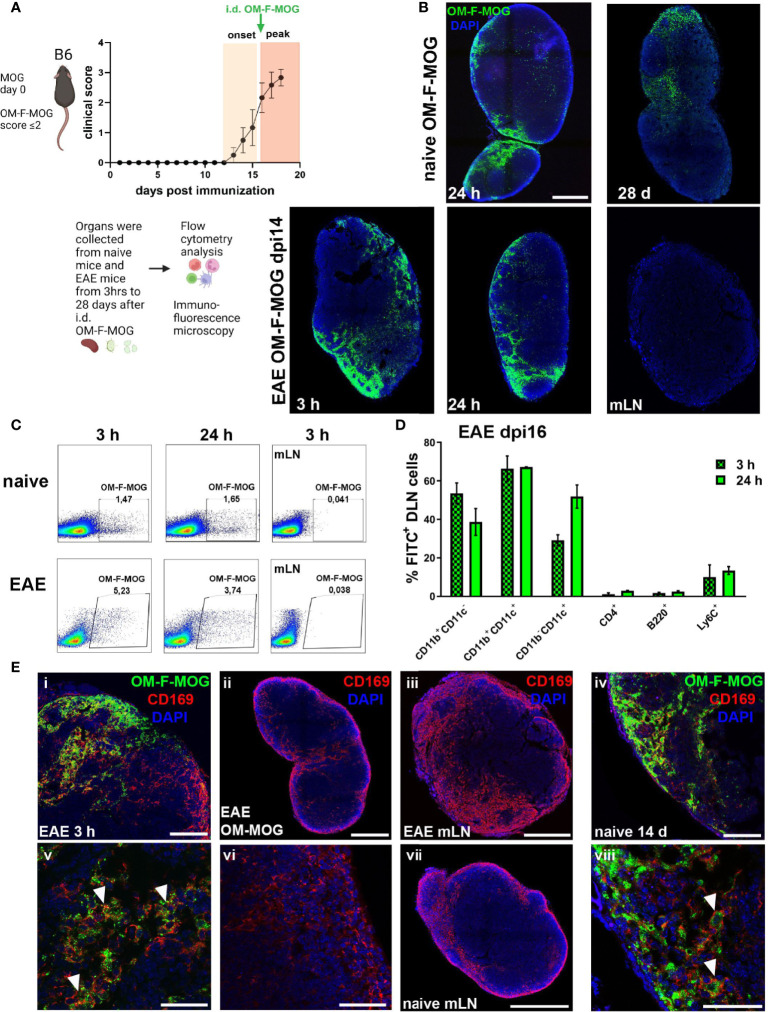
Soluble OM-MOG rapidly localizes to CD169^+^ subcapsular macrophages in the skin DLN: **(A)** Schematic representation of the overall experimental approach and the time point of injection of OM-F-MOG in the EAE mice. **(B)** Frozen sections of draining skin LN (DLN) and mesenteric LN (mLN) recovered from naïve (top row) and EAE mice at dpi 14 (bottom row), after a single i.d. injection of OM-F-MOG, showing the distribution of FITC fluorescence (green) at the indicated time-points and counter-stained with DAPI (blue). Representative images are shown from 1 mouse (naïve) and 1 of 2 mice (EAE). Scale bar 500 μM; 10x objective.**(C)** Flow cytometry plots of DLN cells (first 2 panels in each row) and mLN cells (3^rd^ panels) recovered from naïve and EAE mice at dpi 16, after a single i.d. injection of OM-F-MOG, at the indicated time-points. Representative plots from 1 mouse (naïve) and 1 of 2 mice (EAE). **(D)** Proportions of FITC^+^ cells in different cell populations of the DLN, recovered from EAE mice 3 h and 24 h after a single therapeutic i.d. injection of OM-F-MOG (n=2 mice/time point). **(E)** Frozen sections of DLN and mLN, as shown in **(A)**, immunostained with anti-CD169 (red) and counterstained with DAPI (blue). Tissues were collected from EAE mice at 3 h (i, iii, v) and 24 h (ii, vi), or naïve mice at 14 d (iv, vii, viii), after a single i.d. injection of OM-F-MOG or, as anti-CD169 immunostaining control, OM-MOG without FITC (ii, vi). Scale bars 500 μM, 10x objective (ii, iii, vii); 100 μM; 20x objective (i, iv); 50 μM; 63x objective (v, vi, viii). Representative images from 1 mouse (naïve) and 1 of 2 mice (EAE). Data are from two independent experiments, one for **A, D** (immunohistochemistry), and one for **B, C** (flow cytometry).

To track the biodistribution of OM-MOG after administration i.d. in mice, we designed and synthesized a novel fluorescence-labeled OM-MOG conjugate, in which FITC was linked to the β-Ala-[KG]^5^MOG35-55 peptide prior to its conjugation to OM (OM-F-MOG). Soluble OM-F-MOG was injected i.d. in one hind flank of naïve, preonset-EAE (dpi 10-11), and peak-EAE (dpi 15-16) mice and monitored at different time points in skin draining LN (DLN), distal LN, spleen and PB by flow cytometry and immunofluorescence microscopy (**Figure 6A**). Tracking of OM-F-MOG in both naïve and EAE mice revealed rapid (within 3 h) localization to the subcapsular region of the immediate skin DLN, and not other LN as visualized by fluorescence microscopy, or spleen by flow cytometry (**Figure 6B–D**). Long-term localization was followed in naïve mice up to the last time point studied by fluorescence microscopy (28 days) (**Figure 6B**). FITC fluorescence showed more invasive distribution in skin DLN from EAE mice compared to naïve mice where it was restricted to the subcapsular sinus region (**Figure 6B**). Flow cytometry confirmed rapid and selective localization of OM-F-MOG to the skin DLN in both naïve and EAE mice (**Figure 6B**). OM-F-MOG initially (3 h) colocalized with CD11b^+^CD11c^-^ and CD11b^+^CD11c^+^ populations, and after 24 h also with CD11b^-^CD11c^+^ DC (**Figure 6D**). Double immunostaining with antibodies against CD169 revealed the presence of abundant extracellular FITC immunofluorescence (green) and colocalization with CD169-immunofluorescence in subcapsular sinus macrophages (SSM) (red) from 3 h post-injection in EAE mice (**Figures 6Ei, v**, and not shown). Longitudinal studies in naïve mice showed that OM-F-MOG remained localized to CD169^+^ SSM in skin DLN at 14 days post-injection (**Figure 6E**) and up to the last time point studied (28 days post-injection, not shown).

**Figure 7:**
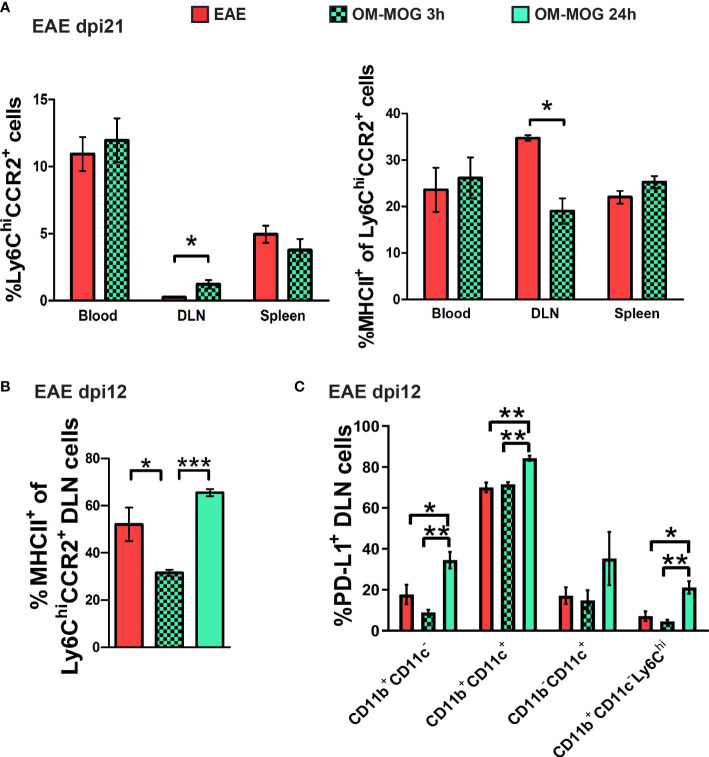
Soluble OM-MOG retains and activates Ly6C^hi^ CCR2^+^ monocytes in the skin DLN: **(A)** Proportions of Ly6C^hi^CCR2^+^ monocytes (left graph), and Ly6C^hi^CCR2^+^ monocytes expressing MHCII (right graph) in blood, spleen and draining inguinal LN (DLN) of EAE mice 3 h after a single therapeutic i.d. injection of vehicle (EAE control) or OM-MOG (n=4/group). **(B)** Proportions of Ly6C^hi^CCR2^+^ monocytes expressing MHCII in the DLN of EAE mice 3 h and 24 h after a single therapeutic i.d. injection of vehicle (EAE control) or OM-MOG (n=3/group). **(C)** Proportions of PD-L1-producing CD11b^+^CD11c^-^, CD11b^+^CD11c^+^ (myeloid DC), CD11b^-^CD11c^+^ (DC) and CD11b^+^CD11c^-^Ly6C^hi^ cells in the DLN of EAE mice 3 h and 24 h after a single therapeutic i.d. injection of vehicle (EAE control) or OM-MOG, as in B (n=3/group). Data and statistical analysis are from one **(B, C)** or one of 2 independent experiments **(A)**. Statistical significance is shown after comparisons between groups using Mann-Whitney test **(A)**, or one-way ANOVA followed by Tukey’s *post hoc* test **(B, C)** (*p ≤ 0.05, **p ≤ 0.01, ***p ≤ 0.001).

We next analyzed the phenotype of Ly6C^hi^ cells recovered from the skin DLN at 3 and 24 h post-injection of OM-MOG in EAE mice by flow cytometry. DLN recovered 3 h post-injection with OM-MOG contained increased proportions of Ly6C^hi^CCR2^+^ cells with reduced MHCII production compared to vehicle controls (**Figures 7A, B**). In contrast, DLN from 24 h post-injection with OM-MOG showed significantly upregulated MHCII and PD-L1 in Ly6C^hi^ cells compared to vehicle controls (**Figure 7B, C**). These results show that i.d. soluble OM-MOG drains directly to the skin DLN, where it targets SSM and locally activates immature Ly6C^hi^ CCR2^+^ myeloid cells to produce MHCII and PD-L1.

### PD-L1 in myeloid cells other than DC is sufficient for OM-MOG immune tolerance and protection against EAE

**Figure 8:**
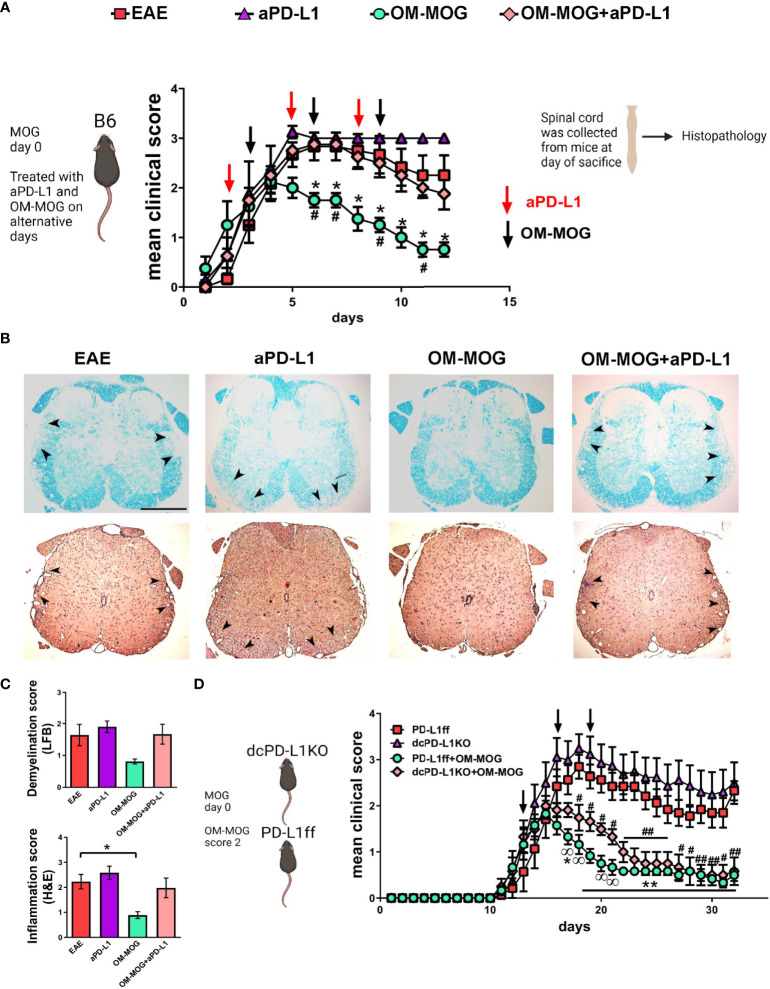
The protective effects of OM-MOG in EAE are abolished by PD-L1 neutralization and not altered by DC-specific PD-L1 knockout. **(A)** Schematic representation of the overall experimental approach and the time points of injection of OM-MOG and anti-PD-L1 neutralizing antibody in the EAE mice. Mean clinical scores of EAE mice treated therapeutically with vehicle (EAE), OM-MOG (black arrows), anti-PD-L1 neutralizing antibody (red arrows), or both OM-MOG and anti-PD-L1 antibody (n=4-6/group). Disease onset was synchronized according to the start of disease in each mouse, occurring between dpi 10-12, so that each mouse was monitored for the same number of days and received the same number of injections. **(B)** Histopathological analysis of spinal cord sections taken from mice shown in **(A)** at the completion of the experiment. Demyelination was measured by Luxol fast blue staining (LFB; upper panels) and inflammatory infiltration by haematoxylin-eosin staining (H&E; lower panels). Confluent inflammatory demyelinating lesions characteristic of EAE are seen in mice treated with vehicle (EAE), anti-PD-L1 antibody and both OM-MOG and anti-PD-L1 antibody (arrowheads), in contrast to markedly reduced lesions in mice treated with OM-MOG. Representative images from 1 of 4 or 6 (EAE) mice/group are shown. Scale bar 500 μM. **(C)** Semi-quantitative analysis of LFB and H&E-stained spinal cord sections of the mice shown in **(A)**. 6-7 sections per mouse were analyzed (n=4-6 mice/group). **(D)** Mean clinical scores of PD-L1ff and dcPD-L1KO EAE mice treated therapeutically with vehicle (EAE) or OM-MOG (black arrows) (n=6-8/group). Data and statistical analysis are from one experiment in each case (one for **A-C**, one for **(D)**. Statistical significance is shown after comparisons between groups using Mann-Whitney test (**A**, *EAE vehicle versus OM-MOG; ^#^OM-MOG vs OM-MOG+anti-PD-L1) (**D**, *PD-L1ff EAE vehicle versus PD-L1ff+OM-MOG; ^#^PD-L1ff EAE vehicle versus dcPD-L1KO+OM-MOG; ^
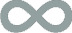
^PD-L1ff+OM-MOG versus dcPD-L1KO+OM-MOG), and one-way ANOVA followed by Tukey’s *post hoc* test **(C)** (*, #, ^
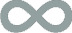
^p ≤ 0.05, **, ## p ≤ 0.01).

To investigate the functional contribution of PD-L1 to the beneficial therapeutic effects of OM-MOG *in vivo*, we administered a neutralizing anti-PD-L1 antibody to EAE mice receiving therapeutic OM-MOG injections. Groups of mice were immunized for the induction of EAE and injected alternatively with a neutralizing antibody to PD-L1 (i.p. 200 μg/mouse) and OM-MOG (i.d.) after the onset of EAE, starting in individual mice when they reached clinical score 2 (**Figure 8A**). As previously reported by others, anti-PD-L1 antibody tended to increase EAE severity, resulting in a chronic non-remitting form of EAE compared to vehicle EAE mice and confirming that PD-L1 is involved in the regulation of EAE (45). As we show in a previous report, therapeutic OM-MOG resulted in steady reduction of clinical symptoms compared to vehicle injected EAE mice (31). In contrast, EAE mice injected with both anti-PD-L1 and OM-MOG developed severe disease equal to that in the vehicle EAE mice, showing that inhibition of PD-L1 is sufficient to abolish OM-MOG immune tolerance. The clinical results were validated by histological analysis of spinal cords recovered from mice at sacrifice on dpi 22 (**Figures 8B, C**). Both vehicle and anti-PD-L1 injected EAE mice showed confluent inflammatory demyelinating lesions in spinal cord white matter. As expected, OM-MOG injected mice showed reduced lesions compared to the vehicle and anti-PD-L1 injected EAE mice. In contrast, mice injected with both OM-MOG and anti-PD-L1 showed confluent inflammatory demyelinating lesions equal to those in control EAE mice.

To investigate the cell source of PD-L1 responsible for OM-MOG tolerance, we next tested OM-MOG immune tolerance in mice with selective deletion of PD-L1 in DC, since DC play critical roles in trafficking antigens to DLN and in presenting antigens to T cells. We crossed mice carrying a LoxP-flanked *Pdl1* allele (PD-L1ff) (37) with mice expressing Cre recombinase under the control of the CD11c promoter to generate DC-specific PD-L1 knockout mice (dcPD-L1KO). PD-L1 production by CD11b^+^CD11c^+^ and CD11b^-^CD11c^+^ DC was equal in spleens recovered from wild type B6 and PD-L1ff mice during EAE (**Supplementary Figure 4B**). PD-L1 production was significantly depleted in corresponding cells from dcPD-L1KO mice (**Supplementary Figure 4B**). We induced EAE in groups of dcPD-L1KO and PD-L1ff controls. Groups of mice of each genotype were injected with OM-MOG starting when individual mice reached clinical score 2. Both dcPD-L1KO and PD-L1ff mice developed full-blown clinical EAE (**Figure 8D**). Protection by OM-MOG showed an initial delay but was thereon equal in dcPD-L1KO and PD-L1ff mice (**Figure 8D**). Together these results show that soluble OM-MOG targets CD169^+^ SSM in DLN, rapidly upregulates PD-L1 production by several myeloid cell populations including Ly6C^hi^ cells, and that OM-MOG tolerance is dependent on PD-L1 production by myeloid cells other than CD11c^+^ DC.

## Discussion

CNS autoimmunity in EAE results from a highly ordered two-way communication between specific auto-reactive helper T (Th) cells and myeloid cells, and during disease development this interaction moves from the periphery into the CNS. Both CD4^+^ T cells and bone marrow derived CD11b^+^Ly6C^hi^ myeloid cells, that expand in the periphery following immunization, are independently critical for the induction and development of EAE (10–12). Effector cell interactions in EAE are a model system for understanding autoimmune components in MS. The design of immunotherapies that would selectively target the immune cells causing disease in MS has mainly focused on myelin-specific T cells, while little attention has been given to myeloid cells. In previous studies we show that OM-conjugated myelin peptides induce robust antigen-specific immune tolerance in EAE, but without altering T cell differentiation to Th1 and Th17 effector cells, except for reduced antigen-specific proliferation responses and some other features of anergy (30, 31). Instead, we show here that OM-MOG targets circulating Ly6C^hi^CCR2^+^ myeloid progenitor cells, inducing their maturation and thereby trapping them in the periphery, and resulting in prevention or treatment of disease depending upon the stage of OM-MOG administration. These results identify a novel mechanism of antigen-specific monocyte-mediated tolerance.

First, using the chemotherapeutic drug GEM to investigate the functional impact of proliferating Ly6C^hi^ and Ly6G^+^ myeloid cells in EAE, as well as in OM-MOG tolerance, we show that depletion of peripheral Ly6C^hi^ and Ly6G^+^ cells by GEM administration prior to disease onset temporarily delays the development of EAE. Recovery of Ly6C^hi^ cells in PB and spleen of GEM-treated mice to levels equal to those in EAE mice coincided with the day of disease onset in the GEM mice, and they developed full-blown EAE thereafter. These results confirm the known role of Ly6C^hi^ cells *via* CCR2 in disease development (10–12). GEM did not reduce immune tolerance by OM-MOG, suggesting that previously described modulatory effects of Ly6C^hi^ and Ly6G^+^ cells in cancer and EAE are not responsible for OM-MOG immune tolerance. Second, the beneficial effects of OM-MOG are associated with selective maintenance of Ly6C^hi^ cells in PB and spleen and prevention of immune cell trafficking to the spinal cord and infiltration during the effector phase; Ly6C^hi^ cell depletion by GEM further prolongs OM-MOG protection. Third, soluble OM-MOG directly drains to skin DLN to be sequestered by CD169^+^ SSM (within 3 h), and locally retains and activates Ly6C^hi^CCR2^+^MHCII^+^ and Ly6C^hi^PD-L1^+^ cells (within 24 h). This is paralleled by reduced immune cell infiltration of the spinal cord and shrinkage of established spinal cord lesions. Fourth, administration of neutralizing anti-PD-L1 antibody in mice with active EAE abolishes the protective effects of therapeutic OM-MOG, while mice with CD11c^+^ cell-specific deletion of PD-L1 remain fully protected, demonstrating a critical role for PD-L1 production by non-DC, possibly Ly6C^hi^ and Ly6C^lo^ myeloid cells and/or SSM, in OM-MOG immune tolerance.

Peripheral Ly6C^hi^ monocytes are known to convert into non-classical alternatively activated macrophages that express high levels of PD-L1 under conditions of myocardial infarction (46). In EAE, Ly6C^hi^ monocytes are required to migrate to the CNS to induce disease, and their conversion to activated macrophages appears to take place within the CNS. Ly6C^hi^ cells show high plasticity within the CNS during EAE, and can become further activated to produce high levels of NO and PD-L1, and acquire MDSC-like properties by suppressing T cell responses, and possibly aid repair (28). Recently the myeloid cell shift from proinflammatory to alternatively activated phenotype was shown to occur at a single cell level in the CNS during the evolution of EAE (15, 19). The relevance for MS was shown by detection of iNOS-expressing inflammatory myeloid cells in the demyelinating rim of chronic active MS lesions whereas alternatively activated macrophages were enriched in the quiescent lesion core (15). We show here that OM-MOG prematurely activates encephalitogenic Ly6C^hi^ monocytes before infiltration into CNS, thereby preventing their migration to the CNS to drive disease. DLN and splenic Ly6C^hi^ myeloid cells showed robustly upregulated production of MHCII and PD-L1 as soon as 24 h after a single skin injection of OM-MOG.

Early studies showed that mannosylated peptide antigens (33, 34) or antigens chemically conjugated to an oxidized form of the yeast polysaccharide mannan (OM) (47), show greatly enhanced presentation by MHCII and MHCI to T cells by inducing the activation of macrophages (48) and maturation of DC (49). Fungal mannan is a pattern recognition receptor (PRR) ligand that induces the maturation of DC *ex vivo*, in a TLR4-dependent manner, thus explaining the adjuvant capacity of OM-conjugated peptide antigens (32). Our finding that soluble OM-MOG rapidly drains from the skin to DLN, localizes to CD169^+^ SSM and results in the recruitment and activation of Ly6C^hi^ myeloid cells is fully supported by a recent study aiming to develop soluble and particulate mannans as an adjuvant strategy (50). There, soluble mannan also rapidly trafficked to DLN to induce a potent innate response characterized by LN expansion, activation of myeloid cells, and upregulated type I and II IFN pathways (50). Until now, the observations that OM-conjugated (30) and mannosylated (35) myelin antigens induce potent antigen-specific immune tolerance in EAE has appeared paradoxical and been difficult to reconcile with the numerous studies of mannans as immune adjuvants. These findings can now be resolved by combining the knowledge that immature Ly6C^hi^ cells are critical for the development of EAE, with our finding here that OM-MOG activates these cells outside the CNS, initially in local DLN, before they migrate to and infiltrate CNS tissues to induce disease. Notably, we previously show that immune tolerance induced by OM-conjugated myelin peptides is exquisitely antigen-specific and associated with the maturation of Th1 and Th17 cells with features of anergy (30). Hence, our results raise the possibility that soluble OM-myelin antigens sequestered in DLN induce immune tolerance to EAE through a double-hit; one by engaging immature Ly6C^hi^CCR2^+^ monocytes resulting in their activation into non-encephalitogenic monocytes; two by delivering a strong Ag-specific reactivation signal to MOG-specific T cells in the context of alternatively activated PD-L1-producing myeloid cells, resulting in T cell anergy.

PD-L1 and PD-L2 are immune checkpoint regulators of the adaptive immune response that act by inducing T cell anergy, cytostasis and apoptosis *via* programmed cell death protein 1 (PD-1) (51). Recent fate mapping studies identified PD-L1 as a specific marker for nonclassical monocytes that are converted from classical Ly6C^hi^ monocytes in PB, spleen and bone marrow. In a myocardial infarction model of inflammation PD-L1 monocytes were clustered in tertiary lymphoid organs and regulated T cell responses (46). Previous studies aiming to induce antigen-specific immune tolerance as a potential immune therapy approach for MS, have shown important roles for PD-L1 and PD-1, including intravenous (i.v.) administration of high-dose free peptide (52), myelin peptide antigens cross-linked to the surface of syngeneic splenic leukocytes with ethylene carbodiimide (ECDI) (53) or encapsulated in nanoparticles (54), and of mannan-conjugated MOG (31). However, details of the cellular & molecular interactions involved, relative involvements of peripheral and central nervous system environments and the role of antigen in tolerance induction in these different strategies are incompletely understood. Recently, high-dose i.v. antigen was shown to halt EAE by inducing PD-L1 expression in monocyte-derived DC in the CNS *via* an IFN-γ (T cell)/IL-27 (DC)-dependent mechanism, and the induction of apoptosis in PD-1^+^ autoreactive T cells (52). In contrast, we show in a previous report that OM-MOG tolerance is associated with the alternative (M2) activation of myeloid cells in the secondary lymphoid organs and not the CNS (31). Also, OM-MOG rapidly induces PD-L1 production by CD11b^+^Ly6C^hi^ and CD11b^+^Ly6C^lo^ myeloid cells and DC in DLN and spleens of mice with ongoing EAE, an effect associated with almost complete amelioration of clinical symptoms and reduction of spinal cord lesions (31). We further show here that PD-L1 is a critical mediator of OM-MOG tolerance because the administration of a neutralizing anti-PD-L1 antibody totally abrogated the therapeutic effects of OM-MOG, while mice with DC-specific deletion of PD-L1 maintained full protection by therapeutic OM-MOG.

In summary, we demonstrate that soluble OM-MOG rapidly localizes to CD169^+^ SSM in skin DLN where it resides over long-term. Its presence in DLN is associated with local alternative activation of PD-L1-producing Ly6C^hi^ cells from EAE-inducing immature Ly6C^hi^CCR2^+^ monocytes (within 24 h), thereby preventing trafficking of monocytes to the CNS to drive disease. Subsequent accumulation of Ly6C^hi^CCR2^+^MHCII^+^ cells in the spleen and blood and disappearance from CNS (within 24 h) is accompanied by the rapid reversal of clinical symptoms and established inflammatory demyelinating lesions in the spinal cord white matter. Tolerance is dependent upon PD-L1 production by non-DC cells, most likely SSM and/or Ly6C^hi^CCR2^+^ cells. As far as we are aware, our studies of OM-conjugated peptides reveal an entirely novel mechanism of antigen-specific peripheral immune tolerance in EAE, in which encephalitogenic myeloid precursor cells and antigen-specific T cells are prevented from trafficking to the CNS to induce inflammation, demyelination and axonal damage. OM-peptides represent a promising approach by which CNS-directed autoimmune responses can be rapidly inactivated over long-term without the need for target tissue intervention and might form the basis for design of novel antigen-specific therapies. The precise molecular interactions that take place in DLN between OM-MOG-containing SSM, immature Ly6C^hi^ myeloid cells and MOG-specific T cells to result in antigen-specific tolerance is the subject of current investigation.

Relevant for the potential translation of fungal mannan-conjugated peptides to humans for the regulation of autoimmune diseases, it has been shown by another group that a OM-conjugated peptide of the tumor protein MUC1 is well tolerated over long-term and showed effectivity in clinical studies in patients with breast cancer (55). In the context of cancer, it is possible that OM-MUC1 also acts by maturing M-MDSC (Ly6C^hi^), which are well known to suppress anti-tumor immune responses, although this possibility needs to be formally tested. In the context of CNS autoimmune disease, the strict antigen-specificity of immune tolerance induced by the OM-myelin peptides in our study suggests that a therapeutic approach would be limited to diseases in which the triggering antigen is known. While the autoimmune targets of CNS-infiltrating T and B lymphocytes in MS are not yet fully understood, the approach might be useful in diseases with known autoantigens such as MOG antibody associated disorders (MOGAD), where MOG is a major candidate autoantigen (56, 57), and neuromyelitis optica spectrum disorders (NMOSD), with an autoimmune response that targets aquaporin-4 on astrocytes, both of which diseases involve infiltrating T and B lymphocytes and inflammatory myeloid cells (57, 58). Our study underscores the importance of peripheral innate immune responses, in this case to a fungal synthetic antigen, in the control of autoimmune demyelination, and raises the possibility that targeted maturation or differentiation of Ly6C^hi^CCR2^+^ inflammatory monocytes in the periphery, outside of the target organ, represents a novel concept for therapy in tissue-specific autoimmune diseases.

## Data availability statement

The original contributions presented in the study are included in the article/**Supplementary Material**. Further inquiries can be directed to the corresponding author.

## Ethics statement

The animal study was reviewed and approved by Committee for Evaluation of Experimental Procedures, Department of Experimental Animal Models, Hellenic Pasteur Institute (Presided by Dr P Andriopoulos pandriopoulos@patt.gov.gr for the Hellenic Republic, General Secretariat for Agricultural Economy, Veterinary and Licenses).

## Author contributions

AD designed and performed prophylactic and treatment EAE experiments, neuropathological, immunohistochemical and imaging experiments, T cell proliferation and flow cytometry analyses, and analyzed results; AP with AD established colonies of PD-L1ff and dcPD-L1KO mice, designed and performed treatment EAE experiments, clinical scoring, immunohistochemistry, flow cytometry experiments and analyzed results; MA designed and performed EAE experiments; AG synthesized, purified and characterized myelin peptide analogues; ME designed and performed splenocyte transfer experiments and analyzed results; M-EA synthesized, purified and characterized myelin peptide analogues; TT designed, synthesized, purified and characterized myelin peptide analogues including the FITC-conjugated OM-MOG peptide; LP was responsible for overall experimental design and management of project, analyzed and interpreted data, wrote paper with help from AD. All authors edited the final version of the paper. All authors contributed to the article and approved the submitted version.

## Funding

This research was co‐financed by European Union and Greek national funds by The Management and Implementation Authority for Research, Technological Development and Innovation Actions (MIA-RTDI/EΥDE-ETAK) of the Hellenic Ministry of Development and Investments, through the Operational Program Competitiveness, Entrepreneurship and Innovation, under the call RESEARCH – CREATE –INNOVATE (project code T1EDK-01859; acronym AKESO).

## Acknowledgments

We thank Arlene Sharpe (Harvard University) for providing the *Pdl1ff* and *Cd11c*-Cre mice, Lydia Sorokin (University of Muenster) for providing the 455 rabbit anti-pan-laminin antibody, Burkhard Becher and Melanie Greter (University of Zurich) for providing CD45.1 congenic B6 mice, and Cecilia Washburn (Sharpe Laboratory, Harvard University) for help and advice with establishment of *Pdl1ff* and *Cd11c*-Cre mouse lines in Athens. Many thanks also to Hellenic Pasteur Institute members Eirini Fragkiadaki, veterinarian, and the other members of the Department of Animal Models for Biomedical Research (DAMBR) for the care of our animals, Evangelia Xingi of the Light Microscopy Unit and FACS Facility, and Maritsa Margaroni, Laboratory of Cellular Immunology, for training and help in use of the FACSAria and confocal microscope, and Fotis Badounas, Trangenic Technology Lab (TTL), for help with whole mount vertebral column histology protocol. AD created schematic abstract and figure schematics using BioRender.com.

## Conflict of interest

M-EA was employed by the company Vianex S.A.

The remaining authors declare that the research was conducted in the absence of any commercial or financial relationships that could be construed as a potential conflict of interest.

## Publisher’s note

All claims expressed in this article are solely those of the authors and do not necessarily represent those of their affiliated organizations, or those of the publisher, the editors and the reviewers. Any product that may be evaluated in this article, or claim that may be made by its manufacturer, is not guaranteed or endorsed by the publisher.

## Abbreviations

B6, C57BL/6 mice; CFSE, carboxyl fluorescein succimidyl ester; DLN, draining lymph node; EAE, experimental autoimmune encephalomyelitis; GEM, gemcitabine; LFB, Luxol fast blue; mLN, mesenteric lymph node; MDSC, myeloid-derived suppressor cells; MHCII, MHC class II; MOG, myelin oligodendrocyte glycoprotein 35-55 peptide; MS, multiple sclerosis; OCT, optimal temperature compound; OM, oxidized fungal mannan; PB, peripheral blood; PFA, paraformaldehyde in PBS; PTx, *Bordetella pertussis* toxin; SSM, subcapsular sinus macrophages.
