# Supplementary figures and images for: Maturation of circulating Ly6ChiCCR2+ monocytes by mannan-MOG induces antigen-specific tolerance and reverses autoimmune encephalomyelitis
Source: Front Immunol. 2022 Sep 9;13:972003. doi: 10.3389/fimmu.2022.972003 (PMC9501702; doi:10.3389/fimmu.2022.972003)

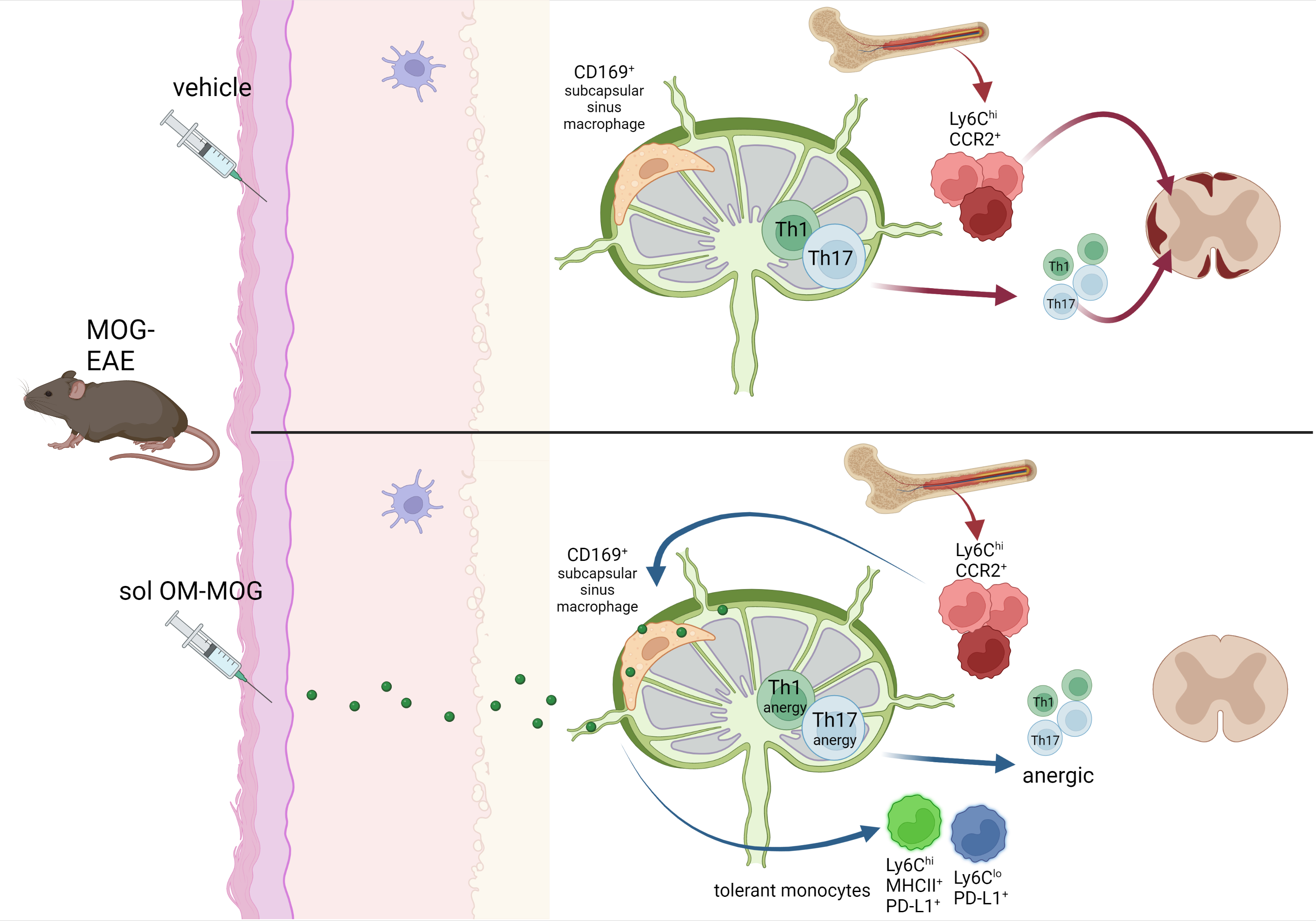

Supplement: Supplementary file 1 [file Image_1.jpg]
