# Supplementary material for: Maturation of circulating Ly6ChiCCR2+ monocytes by mannan-MOG induces antigen-specific tolerance and reverses autoimmune encephalomyelitis
Source: Front Immunol. 2022 Sep 9;13:972003. doi: 10.3389/fimmu.2022.972003 (PMC9501702; doi:10.3389/fimmu.2022.972003)
Supplement: Supplementary file 2 [file DataSheet_1.pdf]

Dagkonaki et al., 2022

Supplemental Material

Supplementary Figure 1

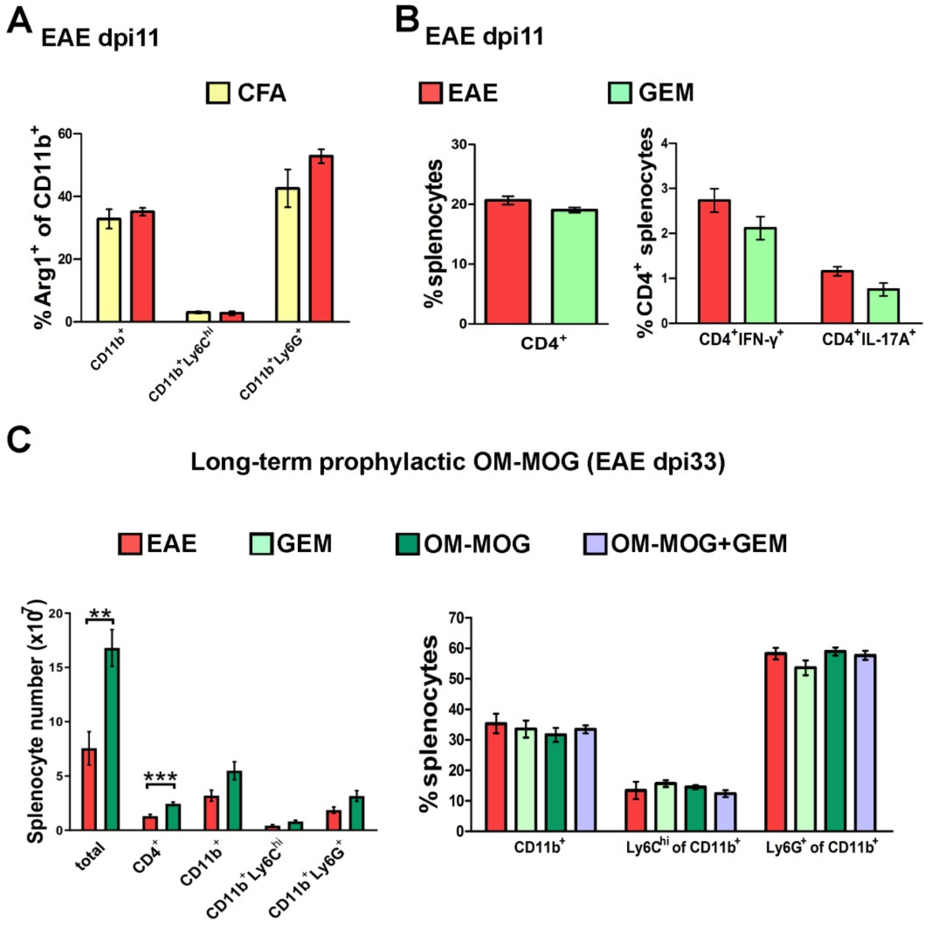

Supplementary Figure 1: **Subpopulations of myeloid cells and T cells in spleens of treated and untreated EAE mice. A)** Proportions of CD11b^{+}, Ly6C^{hi}, Ly6G^{+} myeloid cells producing arginase-1 (Arg1) in spleen of EAE or CFA immunized mice recovered at disease onset in EAE group (dpi 11) (n=3/group). **B)** Proportions of CD4^{+}, CD4^{+}IFN-γ^{+} and CD4^{+}IL-17A^{+} T cells in spleen of EAE mice after pre-onset administration of gemcitabine (GEM), as shown in Fig. 2A, and recovered at disease onset in the EAE group (dpi 11) (n=2-3/group). **C)** Total numbers of splenocytes, CD4^{+} T cells and CD11b^{+}, Ly6C^{hi} and Ly6G^{+} myeloid cells in spleens of EAE mice after long-term prophylactic administration of vehicle or OM-MOG and recovered at sacrifice (dpi 33) (n=3-5/group) (left graph). Proportions of CD11b^{+}, Ly6C^{hi} and Ly6G^{+} myeloid cells in spleens of EAE mice after long-term prophylactic administration of vehicle or OM-MOG, and prophylactic administration of vehicle or GEM as shown in Fig. 2A (dpi 6 & 10) and recovered at sacrifice (dpi 33) (n=3-5/group). Data and statistical analysis are from one experiment for A and B and one for C. Statistical significance is shown after pairwise comparisons between groups using Student's t test (**p≤0.01, ***p≤0.001).

Supplementary Figure 2

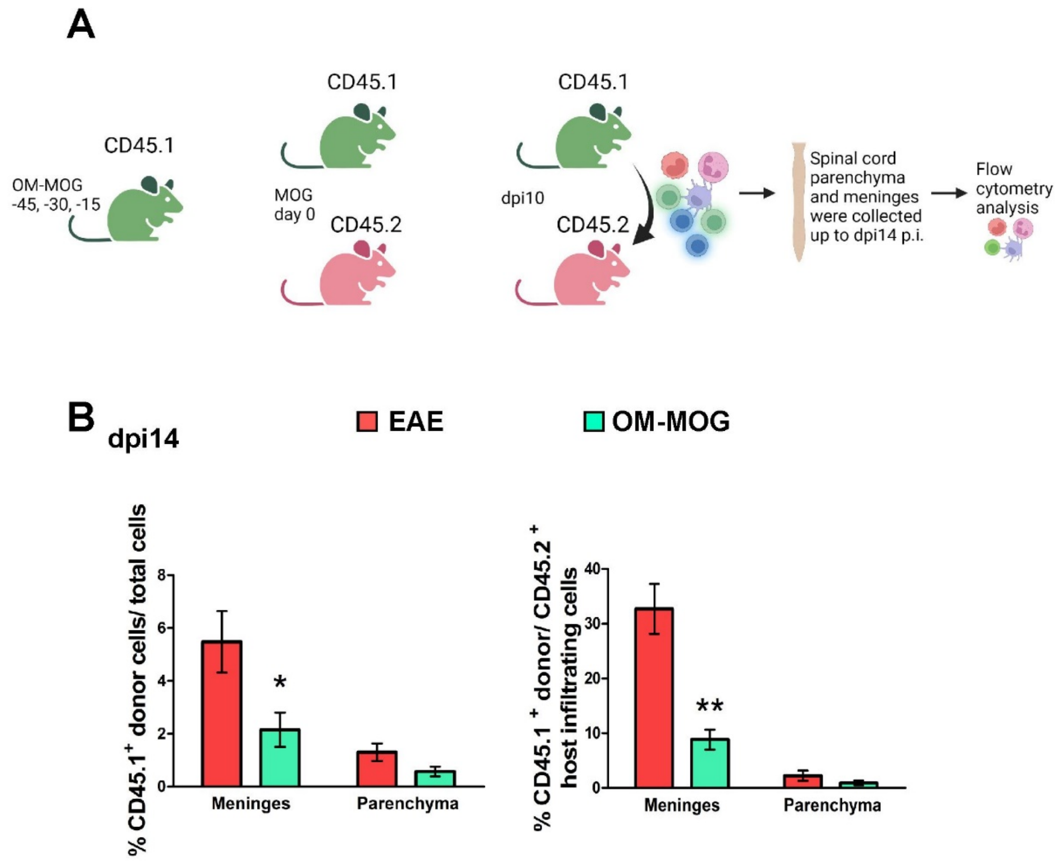

Supplementary Figure 2: Transfer of tolerance by splenocytes from prophylactic OM-MOG mice to EAE recipient mice. A) Schematic representation of adoptive transfer experiment whereby splenocytes and DLN cells isolated from CD45.1 “donor” mice on dpi 9 after long-term prophylactic OM-MOG and induction of EAE (green mice), were adoptively transferred into CD45.2 congenic “recipient” mice on dpi 10 following immunization for EAE. B) Mice sacrificed on dpi 14 (peak) were analyzed for CNS-infiltrating mononuclear cells, separately in spinal cord meninges and parenchyma by flow cytometry (n=3-4/group). Data and statistical analysis are from one representative of two independent experiments. Statistical significance is shown after pairwise comparisons between groups using Student’s t test (*$p \leq 0.05$, **$p \leq 0.01$).

Supplementary Figure 3

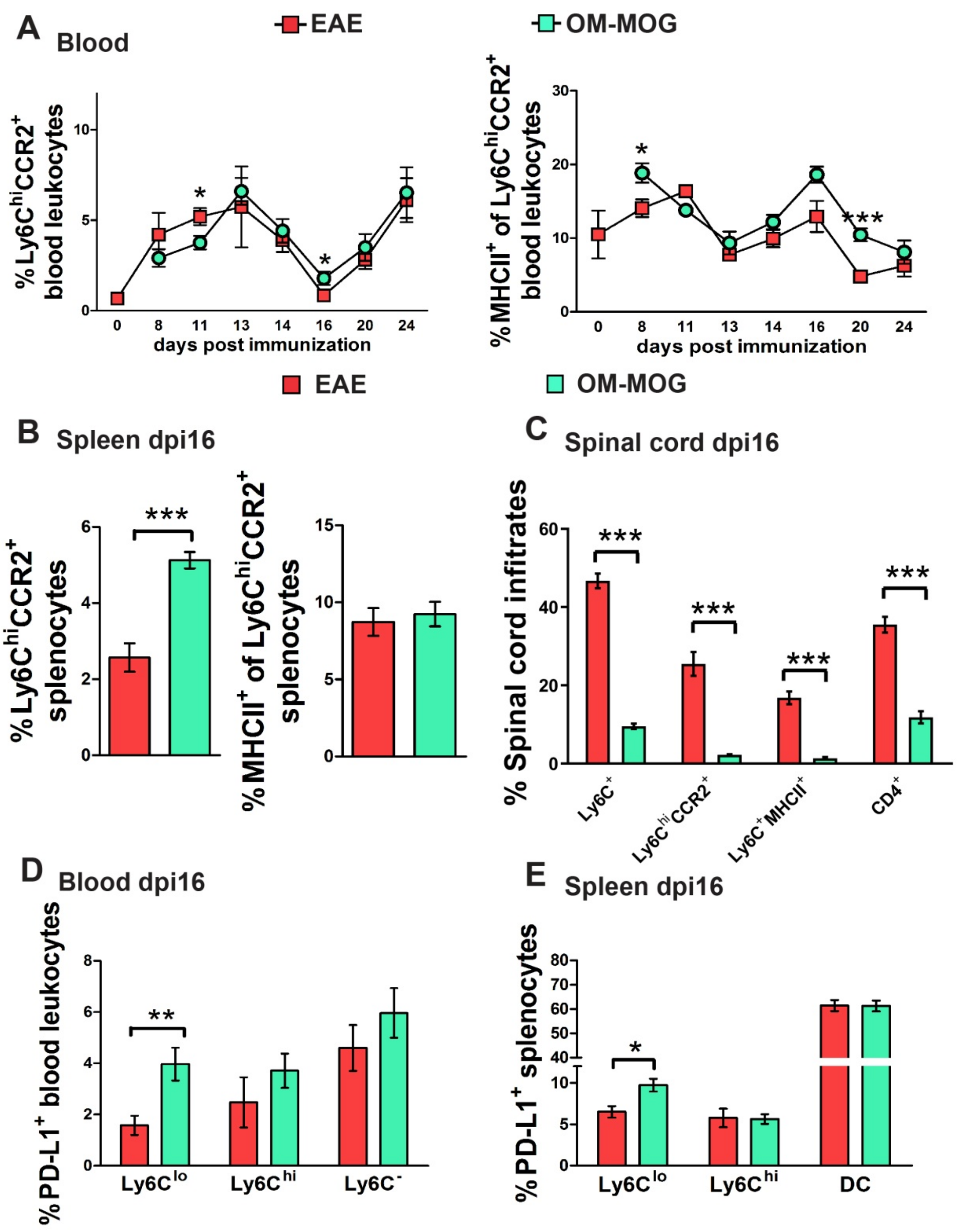

Supplementary Figure 3: Short-term prophylactic OM-MOG retains activated Ly6C^{hi}CCR2^{+}MHCII^{+} myeloid cells in the periphery of EAE mice and inhibits their migration to the spinal cord. **A)** Time-course of proportions of Ly6C^{hi}CCR2^{+} monocytes (left graph) and Ly6C^{hi}CCR2^{+} cells expressing MHCII (right graph) in blood of EAE mice after short-term prophylactic administration of vehicle or OM-MOG (n=3 at day 0 and n=4-10/group/time point). **B)** Proportions of Ly6C^{hi}CCR2^{+} monocytes (left graph) and Ly6C^{hi}CCR2^{+} cells expressing MHCII (right graph) in spleen recovered from groups of mice shown in A at dpi 16 (EAE peak) (n=8 for EAE and n=4 for OM-MOG). **C)** Proportions of Ly6C^{hi}CCR2^{+} and Ly6C^{+}MHCII^{+} myeloid cells and CD4^{+} T cells in CNS-infiltrating mononuclear cells recovered from spinal cord of EAE mice shown in A at dpi 16 (n=8 for EAE and n=4 for OM-MOG). Proportions of PD-L1-producing **D)** CD11c^{-}Ly6C^{lo}, CD11c^{-}Ly6C^{hi} myeloid cells and Ly6C^{-} cells in blood (n=6 for EAE and n=4 for OM-MOG), and **E)** CD11c^{-}Ly6C^{lo}, CD11c^{-}Ly6C^{hi} myeloid cells and CD11c^{+}Ly6C^{-} DC in spleen (n=8 for EAE and n=4 for OM-MOG) of EAE mice as shown in A at dpi 16. Data and statistical analysis are from one experiment. Statistical significance is shown after pairwise comparisons between groups using Student's t test (*p≤0.05, **p≤0.01, ***p≤0.001).

Supplementary Figure 4

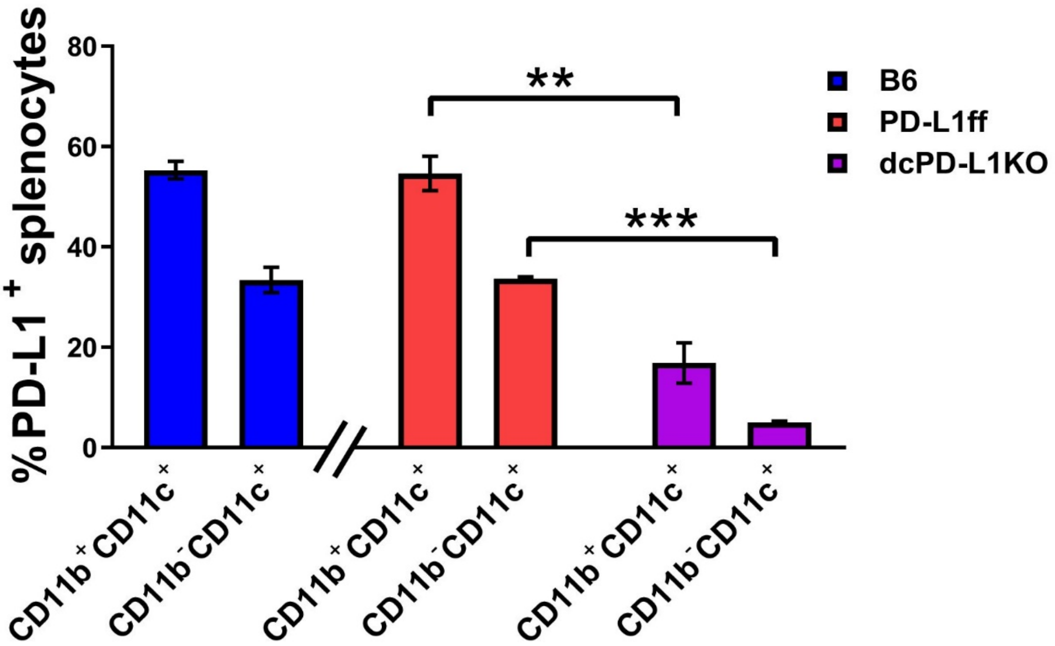

Supplementary Figure 4: Efficiency of PD-L1 depletion in DC of dcPD-L1KO mice. Proportions of PD-L1 producing $CD11b^{+}CD11c^{+}$, $CD11b^{-}CD11c^{+}$ DC populations in the spleens of B6, PD-L1ff and dcPD-L1KO mice, recovered in the chronic phase of EAE (n=3-4/group). Data and statistical analysis are from one experiment. Statistical significance after comparisons between groups using Student's t test is shown (**$p \leq 0.01$, ***$p \leq 0.001$).

Supplemental Methods

Synthesis of linear FITC-β-Ala-[Lys-Gly]$_5$MOG$_{35-55}$ (Ser^{42}) peptide (F-MOG)

FITC-β-Ala-Lys-Gly-Lys-Gly-Lys-Gly-Lys-Gly-Lys-Gly-Met-Glu-Val-Gly-Trp-Tyr-Arg-Ser-Pro-Phe-Ser-Arg-Val-Val-His-Leu-Tyr-Arg-Asn-Gly-Lys-OH

The linear FITC-β-Ala-[Lys-Gly]$_5$MOG$_{35-55}$ (Ser^{42}) peptide (F-MOG) was synthesized in solid phase by 9-fluorenylmethylocarboxyl/t-butoxy (Fmoc/tBu) methodology with the use of Fmoc protected amino acids, 2-chlorotrityl chloride (CLTR-Cl) resin and the standard *N,N'*-diisopropyl carbodiimide/1-hydroxybenzotriazole (DIC/HOBt) coupling reagents (1) (Diagram 1). The first N^a-Fmoc protected amino acid (1.5 mmol) was coupled via esterification to the resin (1 g resin for each peptide) in the presence of diisopropylethylamine (DIPEA) (4.5 mmol/g of resin) in dichloromethane (DCM) (10ml/g of resin) for 1.5 h at room temperature (RT). Subsequently DIPEA (0.5 ml/g of resin) and methanol (MeOH) (1 ml/g of resin) were added in the mixture under stirring at RT for 30 min. A mixture of DCM/MeOH/DIPEA (80:15:5) (10 ml/g of resin) was used to bind the remaining active site of the resin (3 washes for 5, 10 and 15min). Afterwards, the resin was washed with dimethylformamide (DMF) followed by the Fmoc deprotection with a solution of 25% piperidine in DMF (3 washes for 5, 10 and 15 min). The remaining peptide chain was assembled by sequential couplings of the following Fmoc protected amino acids in the presence of DIC and HOBt in dimethylacetamide (DIMAC) followed by Fmoc removal with 25% piperidine solution. The completeness of each coupling and deprotection step was verified by thin layer chromatography (TLC) using AcN/H_2O (2:1) as eluent systems and Kaiser test. The conjugation FITC was achieved after the removal of Fmoc group by the *N*-terminal amino acid (β-Ala). FITC (x3 eq. based on the resin substitution) was dissolved in DMF and then DIPEA (x6 eq.) and the mixture was incubated overnight in the dark at RT. The solution was removed and the resin was washed with DCM. The protected peptide was cleaved from the resin by treatment with DCM/TFE (2,2,2-trifluoroethanol) (7:3) for 2 h in the dark at RT and then treated with the appropriate deprotection solution

[trifluoracetic acid (TFA) / water / 1,4 dithiothreitol (DTT) / thioanisole (92 / 3 / 1 / 4)] for 6 h in the dark at RT. The crude deprotected peptide was purified by semi-preparative reverse phase high performance liquid chromatography (RP-HPLC) and identified by electron spray ionization mass spectrometry (ESI-MS). The purity of the final product (labeled: F-MOG) was verified by RP-HPLC. Scheme 1 shows the synthetic procedure of the F-MOG peptide.

Conjugation of F-MOG peptide with mannan (OM-F-MOG)

The conjugation of F-MOG with mannan was achieved using the described previously methodology (2). 14 mg of mannan from *Saccharomyces cerevisiae* (Sigma Aldrich, Athens, Greece) was dissolved in 1 ml phosphate buffer pH 6.0. Mannan was oxidized to polyaldehyde after treating with 0.01M sodium periodate for 1 h at 4 °C and then ethanodiol was added for 30min at 4° C. OM was purified by size exclusion chromatography (Sephadex G-25M column, Pharmacia Biotech, Sweden), equilibrated with bicarbonate buffer pH 9.0 and the fraction containing the oxidized mannan was collected. Two (2) ml of the selected oxidized mannan in bicarbonate buffer pH 9.0 reacted with 1 mg of F-MOG peptide for 48 h in the dark at RT. The completeness of conjugation was determined by tricine sodium dodecyl sulfate–polyacrylamide gel electrophoresis (SDS-PAGE) as previously described (2).

Characterization of OM-F-MOG

The purification of the labeled peptide was achieved by semi-preparative HPLC and peptide purity was verified by analytical RP-HPLC (Fig. 1A, B). Analytical RP-HPLC for the purified and lyophilized labeled peptide showed only one peak eluting at 10.1 min (Fig. 1B). Identification of the F-MOG peptide was assessed by ESI-MS (Fig. 1C). The molecular mass is 3964 Da and the ESI-MS showed the following peaks: M+3H+/3=1323, M+4H+/4=992, M+5H+/5=794, M+6H+/6=662

The confirmation of the conjugation reaction between F-MOG and OM was achieved by SDS-PAGE, followed by Coomassie staining. The free peptide was detected as a band with

molecular weight 3.0-3.5 kDa, and the conjugate of OM with the labeled peptide (OM-F-MOG) appeared as diffuse smear with molecular weight of 20-200 kDa. The diffuse smear of the conjugate is because of the heterogeneity of the mannan moiety containing different number of mannose units both in the main and the side chains. The fact that the band at 3.5 kDa in the conjugate was missing, confirmed that the conjugation was complete.

References

1) Ieronymaki M, Androutsou ME, Pantelia A, Friligou I, Crisp M, High K, Penkman K, Gatos D, Tselios T. Use of the 2-chlorotrityl chloride resin for microwave-assisted solid phase peptide synthesis. *Biopolymers* (2015) 104(5): 506-514. doi: 10.1002/bip.22710

2) Tapeinou A, Androutsou ME, Kyrtata K, Vlamis-Gardikas A, Apostolopoulos V, Matsoukas J, Tselios T. Conjugation of a peptide to mannan and its confirmation by tricine sodium dodecyl sulfate-polyacrylamide gel electrophoresis. *Anal Biochem* (2015) 485: 43-45. doi: 10.1016/j.ab.2015.06.010

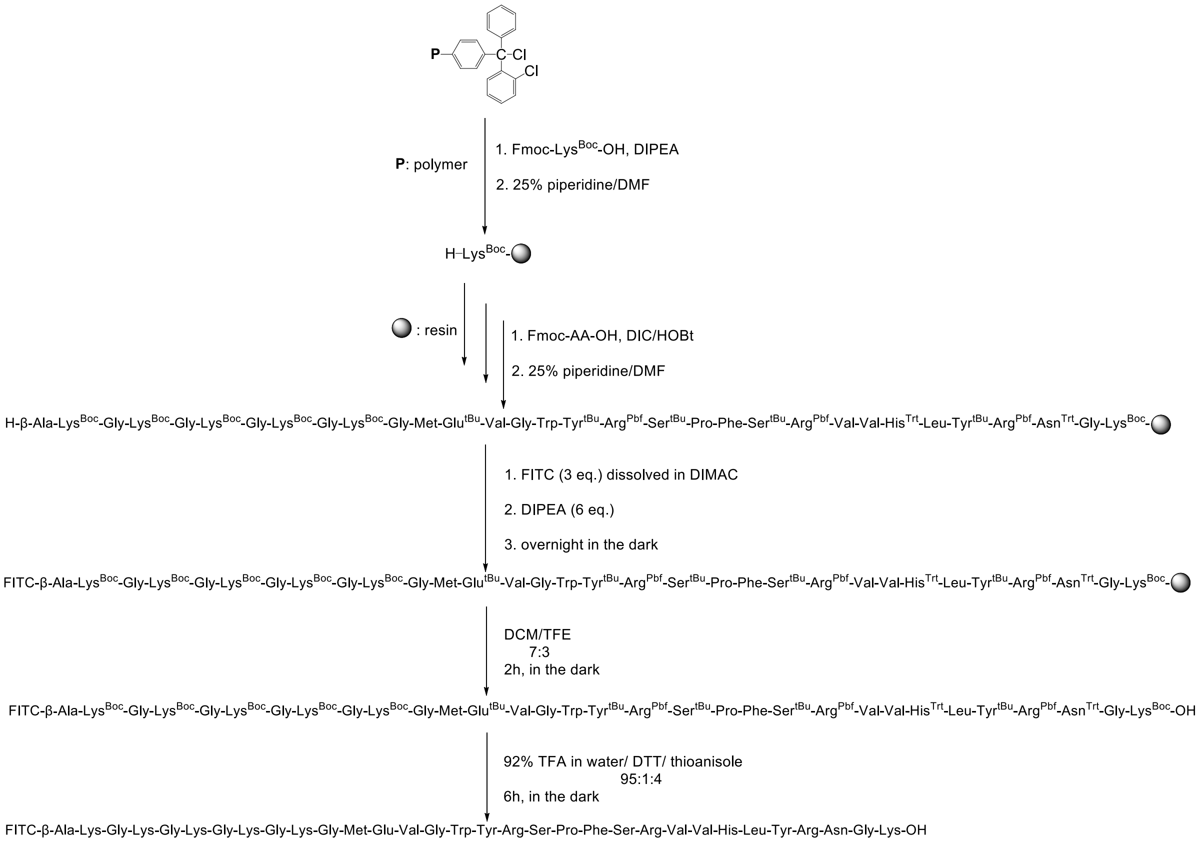

Diagram 1: Synthetic procedure of F-MOG peptide.

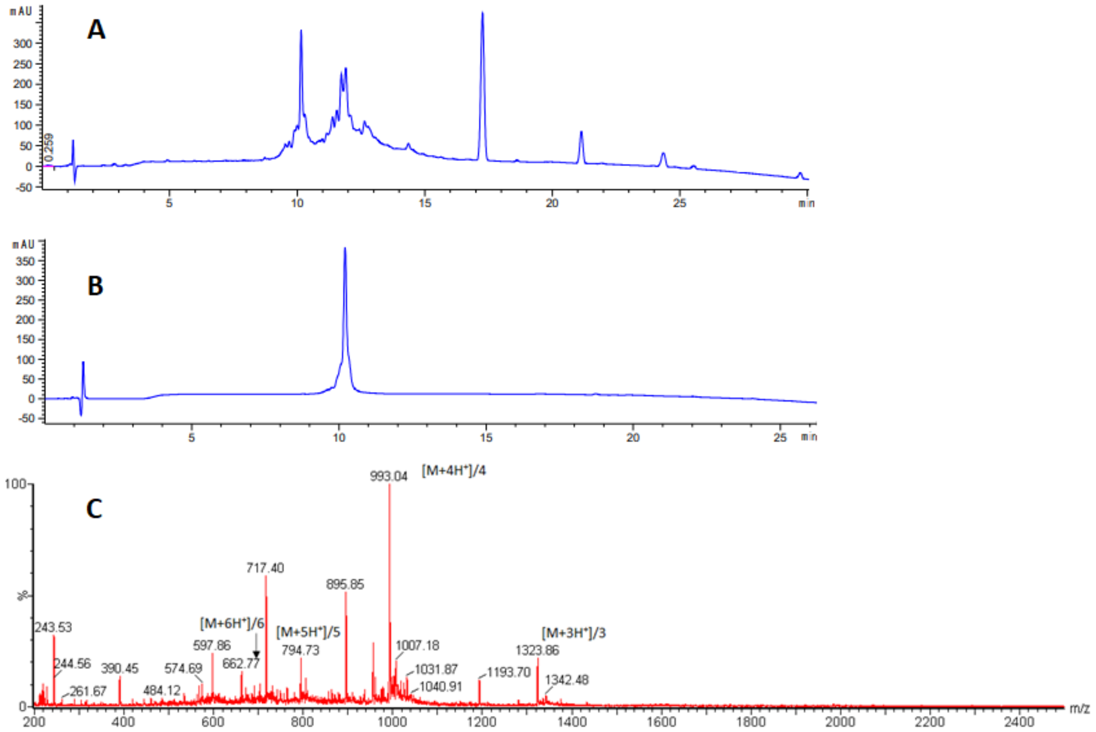

Diagram 2: Representative analytical Reverse Phase High Performance Liquid Chromatography (RP-HPLC) chromatograms of crude (A) and purified (B) F-MOG peptide at 214.4nm using the 1260 Infinity, Quaternary Pump VL Agilent HPLC Instrument and Agilent 1260 Diode Array Detector. RP-HPLC Conditions: i) Temperature: 25 oC, ii) Column: Agilent ZORBAX Eclipse Plus C18 column (3.5μm, 100x4.6mm), iii) Solvents: H2O (0.08% TFA), ACN (0.08%TFA), iv) Gradient elution: from 10% ACN to 100% ACN over 30min. Identification of synthesized peptide using ESI-MS (C).
